# Improving hindlimb locomotor function by Non-invasive AAV-mediated manipulations of propriospinal neurons in mice with complete spinal cord injury

**DOI:** 10.1038/s41467-021-20980-4

**Published:** 2021-02-03

**Authors:** Benedikt Brommer, Miao He, Zicong Zhang, Zhiyun Yang, Jessica C. Page, Junfeng Su, Yu Zhang, Junjie Zhu, Emilia Gouy, Jing Tang, Philip Williams, Wei Dai, Qi Wang, Ryan Solinsky, Bo Chen, Zhigang He

**Affiliations:** 1 F.M. Kirby Neurobiology Center, Boston Children’s Hospital, and Departments of Neurology and Ophthalmology, Harvard Medical School, Boston, MA USA; 2 Department of Ophthalmology and Visual Sciences, Washington University School of Medicine, St. Louis, MO USA; 3 Spaulding Rehabilitation Hospital, Boston, MA USA; 4 Department of Physical Medicine and Rehabilitation, Harvard Medical School, Boston, MA USA; 5 Department of Neuroscience, Cell Biology, & Anatomy, University of Texas Medical Branch, Galveston, TX USA

**Keywords:** Neuroscience, Physiology

## Abstract

After complete spinal cord injuries (SCI), spinal segments below the lesion maintain inter-segmental communication via the intraspinal propriospinal network. However, it is unknown whether selective manipulation of these circuits can restore locomotor function in the absence of brain-derived inputs. By taking advantage of the compromised blood-spinal cord barrier following SCI, we optimized a set of procedures in which AAV9 vectors administered via the tail vein efficiently transduce neurons in lesion-adjacent spinal segments after a thoracic crush injury in adult mice. With this method, we used chemogenetic actuators to alter the excitability of propriospinal neurons in the thoracic cord of the adult mice with a complete thoracic crush injury. We showed that activating these thoracic neurons enables consistent and significant hindlimb stepping improvement, whereas direct manipulations of the neurons in the lumbar spinal cord led to muscle spasms without meaningful locomotion. Strikingly, manipulating either excitatory or inhibitory propriospinal neurons in the thoracic levels leads to distinct behavioural outcomes, with preferential effects on standing or stepping, two key elements of the locomotor function. These results demonstrate a strategy of engaging thoracic propriospinal neurons to improve hindlimb function and provide insights into optimizing neuromodulation-based strategies for treating SCI.

## Abstract (web summary)

After complete spinal cord injury, spinal segments below the lesion maintain inter-segmental communication via the intraspinal propriospinal network. Here, the authors show that neurons in these circuits can be chemogenetically modulated to improve locomotor function in mice after spinal cord injury.

## Introduction

It is known that the spinal cord possesses intrinsic neural circuits, often referred to as central pattern generators (CPGs), that are sufficient to generate many types of behaviourally important motor commands^1,2^. Yet the question remains as to why such CPG networks do not mediate functional restoration in patients and animal models of severe spinal cord injury (SCI). A suggested culprit is the substantially reduced excitability of the spinal network after injury^3–6^. Thus, a logical repair strategy is to develop methods to re-engage CPGs for functional restoration^7–9^. In intact animals, CPG neurons in the lumbar spinal cord receive brain-derived commands and peripheral sensory afferent inputs, as well as signals from intraspinal/propriospinal neurons that are distributed throughout all spinal levels^10–15^. After a complete injury at upper levels, lumbar CPG neurons are deprived of descending inputs from the brain but are still directly or indirectly connected with both propriospinal networks and peripheral sensory afferents below the lesion. Several methods, such as epidural electrical stimulation and/or rehabilitative training, and pharmacological treatments have been shown to confer improved locomotor function in animal models and patients with severe SCI^8,16–18^. However, the achieved recovery is variable, often limited, and without clearly defined functional mechanisms. Thus, there is a pressing need to understand how such spinal CPGs are regulated after losing supraspinal inputs and identify more precise targets that mobilize local spinal circuitry, particularly for treatments like electrical stimulation.

In principle, lumbar CPG neurons and/or their remaining inputs, both sensory afferents and propriospinal networks, could be potential targets for re-activating CPGs for functional restoration^5,19^. However, whether any of these components underlie the functional outcomes of electrical stimulation and/or rehabilitation training remains an intensively debated topic^5,16,19^. In the case of propriospinal neurons, whose axons form intricate networks among different spinal segments, several studies demonstrate that they form indirect pathways relaying brain-derived signals to the spinal cord in conditions of incomplete SCI^20–22^. However, it is unknown whether these neurons, in the absence of brain-derived commands, are sufficient to initiate behaviourally relevant activity patterns in lumbar CPGs.

To address these questions, we focused on adult mice with complete crush injury at thoracic levels, as manipulating either propriospinal neurons with descending projections or neurons in the lumbar spinal cord could serve as a good starting point. Different from the concentrated location of lumbar CPG neurons, propriospinal neurons are widely dispersed at all spinal levels. Thus, a technical challenge is how to efficiently target these propriospinal neurons. Direct injection of viral vectors to the SCI site has been used to express neurotrophic factors^23,24^. However, such an invasive procedure needs multiple injections, has variable efficiency, and could further traumatize the overlying structure of the spine as well as the remaining spinal cord tissue adjacent to the lesion site. On the other hand, following trauma to the spinal cord, the blood-spinal cord barrier (BSCB) breaks down due to vascular damage^25^ and potential secondary inflammation, thereby remaining temporarily impaired. We reasoned that AAV transduction of the SCI penumbra would be possible through intravenous administration during the acute phases after injury. In this study, we first systematically analyzed the transduction efficiency and the cellular tropism of ten different AAV serotypes after intravenous injection at different time points post-SCI. We used our optimized method to express chemogenetic actuators, either the excitatory or inhibitory version of Designer Receptors Exclusively Activated by Designer Drugs (DREADDs), in different populations of thoracic neurons around the lesion and demonstrated that different manipulations led to strikingly distinct motor function improvements. Our results demonstrate the feasibility of mobilizing the intrinsic lumbar neural circuitry for functional restoration by propriospinal axon-assisted remote control.

## Results

### Tail vein injected AAV2/9 efficiently transduces the neurons around lesions in the thoracic spinal cord

**Fig. 1:**
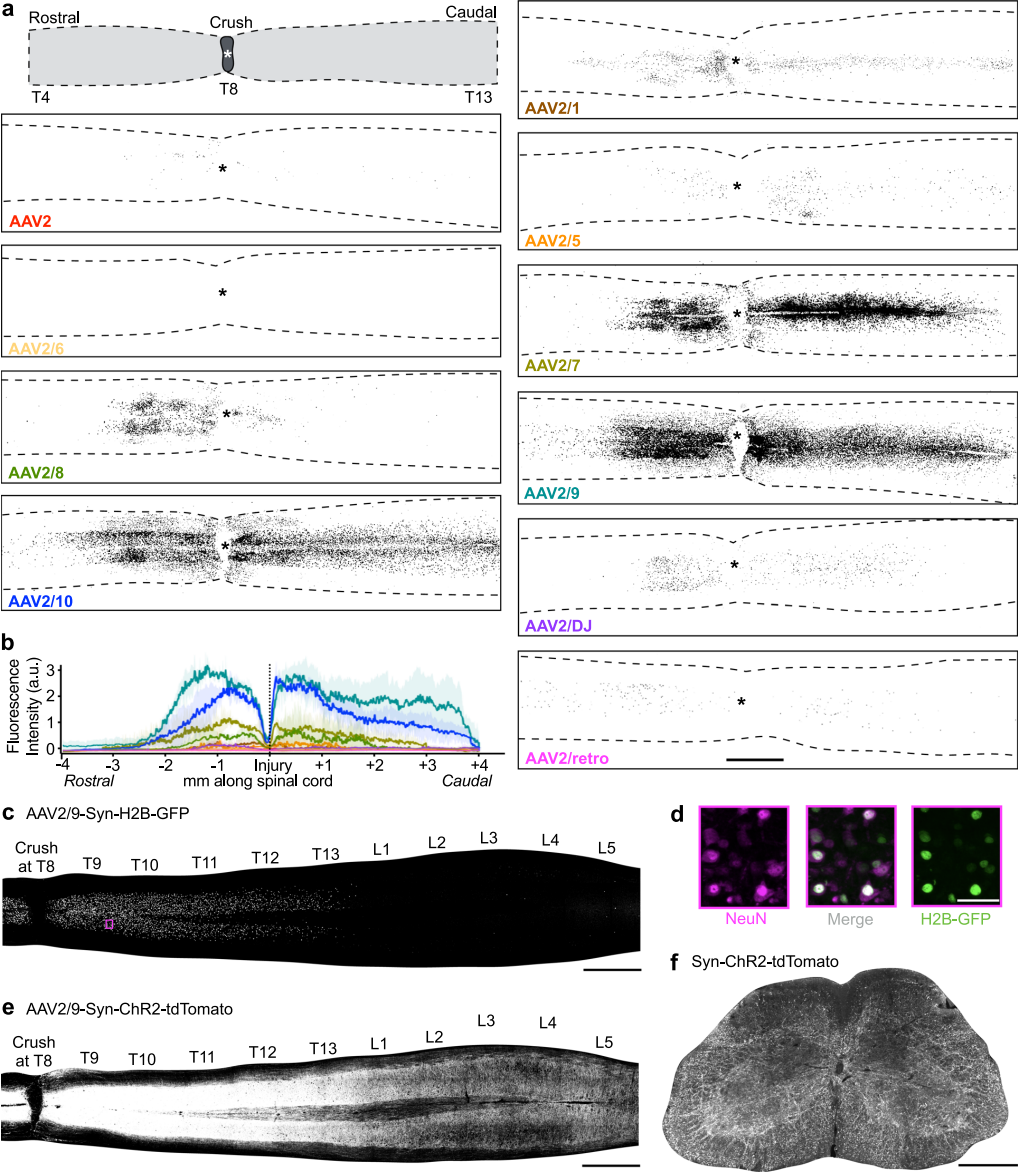
A screen for AAV serotypes that are able to cross blood spinal cord barrier and transduce cells around the lesion after spinal cord crush. **a** Representative images of longitudinal sections of the thoracic spinal cord show transduced cells after tail vein injection of different serotypes of AAVs expressing CAG-H2B-GFP at 3 h after T8 crush injury. Similar distributions were observed in all of 3 analysed mice for each AAV serotype. Scale bar: 1 mm. An asterisk represents lesion centre. **b** Quantification of GFP expression along the longitudinal axis. Mean ± SD are indicated by line and shadow with corresponding colours. a.u.: arbitrary unit. The fluorescence intensities were normalized to that in AAV2/9 (*n* = 3 mice for each AAV serotype, 5 sections per mouse). **c** Representative image showing the expression of H2B-GFP (cell bodies only) in different spinal levels. Similar distributions were observed in all of 3 analysed mice. Scale bar: 1 mm. **d** Magnification of the highlighted area one spinal segment away from the lesion (purple box in (**c**)), indicating co-localization of H2B-GFP and NeuN. Scale bar: 100 µm. **e** Representative image showing the expression of ChR2-tdTomato (cell bodies and axons) in different spinal levels. Similar distributions were observed in all of 3 analysed mice. Scale bar: 1 mm. **f** Cross section of the lumbar spinal cord showing descending ChR2-tdTomato+ axons from thoracic propriospinal neurons. Similar distributions were observed in all of 3 analysed mice. Scale bar: 500 µm.

We first attempted to develop a method to selectively target neurons, including descending projecting propriospinal neurons in the thoracic levels around the lesion site. Since SCI leads to an acute breakdown of the BSCB, we reasoned that intravenous administration of AAVs could yield efficient transduction of cells surrounding the lesion site without surgical intervention. To determine which AAV serotypes could best transduce cells around the lesion epicentre, we injected 8•10^12^ gc particles of AAV-CAG-H2B-GFP, packaged within different serotypes, into the tail vein of mice 3 h after a T8 complete crush injury (Fig. 1a). CAG is a ubiquitously expressed promoter, and H2B-GFP, a histone 2B-GFP fusion protein, is localized in the nuclei of transduced cells, facilitating the assessment of transduction efficiency. We chose 3 h after injury to represent the earliest clinically feasible time point post SCI for such a treatment. The serotypes tested included 2/1, 2, 2/5, 2/6, 2/7, 2/8, 2/9, 2/10, retro-AAV, and DJ. We found that serotypes 2, 2/5, and 2/6 showed almost no expression; all other serotypes led to appreciable transduction of cells restricted to the area adjacent to the lesions examined at 14 days after injection (Fig. 1a, b). Among them, serotypes 2/9 and 2/10 achieved the strongest expression across approximately two spinal cord segments (Fig. 1a, b).

While the experiments above were performed with viral injection at 3 h after injury, we next assessed the duration of this time window for vein injected AAV vectors to cross the BSCB and transduce cells around the lesion after injury. To do this, we focused on AAV2/9, which is known to have limited ability of crossing the BBB in the intact adults and AAV2/7 (Supplementary Fig. 1a). We injected H2B-GFP vectors of these serotypes into the tail vein of mice at different time points (0 or 3 h, and 1, 3, or 7 days) post injury. As shown in Supplementary Fig. 1a–d, GFP expression remained strong when tail vein injection was performed 1 day after crush, but gradually reduced when injected 3 days after SCI and was barely detectable when introduced at 7 days after injury.

To characterize the tropism of tail-vein injected AAV-H2B-GFP vectors in injured spinal cords, histological spinal cord sections were immunostained with markers of individual cell types and then subjected to a custom-made quantification algorithm (see Methods for details). AAV2/9 primarily transduced neurons followed by oligodendrocytes and astrocytes but not pericytes (PDGFRβ), microglia/macrophages (CD68), or endothelial cells (CD31) (Supplementary Fig. 1e–i).

To assess whether such transduced cells include propriospinal neurons with descending projections, we co-injected AAV2/9-Syn-H2B-GFP (to visualize the cell bodies of transduced neurons) and AAV2/9-Syn-ChR2-tdTomato (to visualize both the axons and cell bodies of transduced neurons, including propriospinal axons) to the tail vein of adult mice at 3 h after injury. As shown in Fig. 1c, the transduced neuronal somas (GFP+) are almost exclusively localized in thoracic levels around the lesion site. Importantly, numerous tdTomato+ axons were detected in the lumbar spinal cord (Fig. 1e, f), suggesting efficient labelling of propriospinal neurons with descending projections to the lumbar spinal cord.

### DREADD-assisted activation of thoracic neurons leads to hindlimb locomotor improvements after T8 complete crush injury

With the AAV2/9-based system to target propriospinal and other neurons around the lesion site with high efficiency, we then used this method to express chemogenetic actuators in these neurons and examined the behavioural outcomes of altering their excitability in mice with SCI. To do this, we focused on adult mice with T8 complete crush injury, a surgical procedure that destroys all neural tissues at the lesion level^22,26,27^. To further characterize this injury model, we monitored serotonergic axons around the lesion at different time points by immunohistochemistry with antibodies against 5-HT (Supplementary Fig. 2), a surrogate marker for completeness of injury. This method was used to label all serotonergic axons, thus bypassing issues of partial labelling observed by most tracing procedures^28^. As shown in the serial section of a single mouse spinal cord, as well as quanification of those from different mice (Supplementary Fig. 2a, d), no 5-HT axons could be detected beyond the lesion acutely after injury, supporting the completeness of this injury model. However, at 8 weeks after injury, a few axons were seen at the spinal cord within 1–2 mm distal to the lesion (Supplementary Fig. 2b, d), consistent with previous studies showing spontaneous regrowth of serotonergic axons^28–30^. Thus, we used this model to assess the effects of manipulating propriospinal neurons on hindlimb motor function in the absence of supraspinal inputs.

**Fig. 2:**
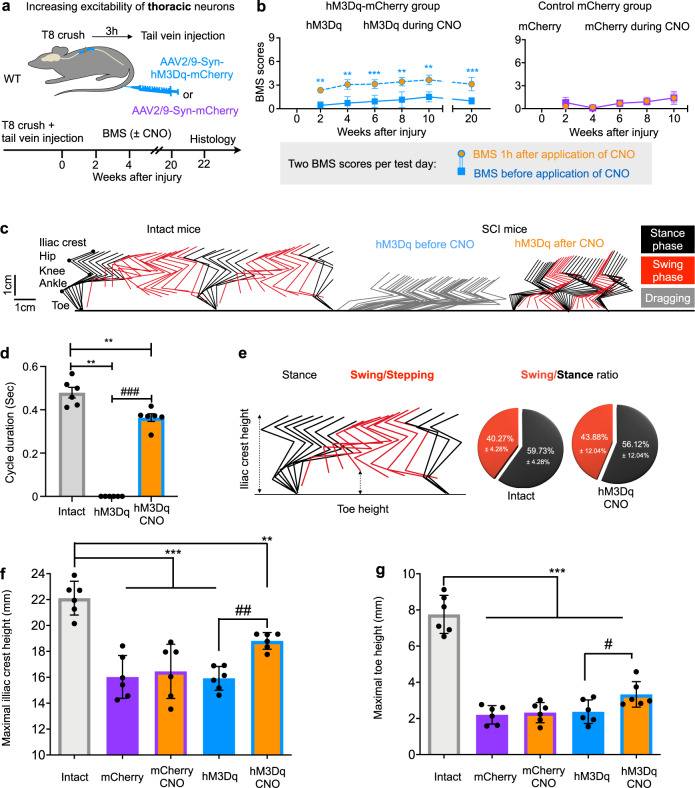
hM3Dq-assisted activation of thoracic neurons elicits locomotion behaviour after T8 crush. **a** Experimental schematic. After a T8 crush injury, wild type (WT) mice received a tail vein injection of AAV2/9-Syn-hM3Dq-mCherry or control virus (AAV2/9-Syn-mCherry) and their locomotion behaviour was measured upon CNO administration every 2 weeks after injury. **b** BMS scores of injured mice with AAVs expressing hM3Dq-mCherry (left) or control mCherry (right) at indicated time points, before and 1 h after administration of 1 mg/kg CNO. hM3Dq-mCherry group: *n* = 7; Control group: *n* = 5. Data shown as mean ± SD (and also for other panels). Factorial Repeated Measures ANOVA with 2 within-subjects factors, followed by two-tailed paired *t* test at each time point. ns: no statistical difference, **p = 0.0090 (2 weeks); 0.0081 (4 weeks); 0.0012 (8 weeks); 0.0029 (10 weeks), ***p < 0.0001 (6 weeks, 20 weeks). **c** Stickviews of kinematic hindlimb analysis of intact mouse and mouse with hM3Dq expression before and after CNO injection (1 mg/kg) at 4 weeks after injury. The phases of dragging, stance, or swing were marked in indicated colours. Cycle duration (**d**) and swing/stance ratio (**e**) of intact mice and spinal cord injured mice with hM3Dq expression. Injured mice expressing hM3Dq dragged before CNO treatment, but had similar stance/swing phase distributions as intact mice (Bar chart) after CNO treatment. Two tailed paired *t* test for within-group comparison (#) and Wilcoxon rank sum for between-group comparison (*). **p = 0.0022 (hM3Dq); 0.0043 (hM3Dq CNO) and ^###^*p* < 0.001. Quantification of the maximum iliac crest heights (**f**) and the maximum toe heights (**g**). One-Way ANOVA with Tukey’s post test for between-group (*) and two tailed paired *t* tests for within-group (#) comparsion. ***p < 0.001; **p = 0.0039; ^##^*p* = 0.0040 (**f**); ***p < 0.001; ^#^*p* = 0.0166. For (**d**)–(**g**), *n* = 6 for each group.

To alter neuronal excitability, we used chemogenetic actuators, either activating hM3Dq or inhibiting hM4Di DREADDs^31^, under control of human synapsin promoter (Syn) to drive neuron-specific expression. Wild-type adult mice were subjected to a T8 complete crush injury followed by a tail vein injection of AAV2/9-Syn-hM3Dq-mCherry or its control (AAV2/9-Syn-mCherry) 3 h after injury (Fig. 2a). Hindlimb locomotor performance was assessed by the Basso Mouse Scale (BMS), an established open-field locomotion test with scores ranging from 0 (no movement of hindlimbs, complete paralysis) to 9 (coordinated plantar stepping in intact mice)^32^. As shown in Fig. 2b, this injury caused hindlimb paralysis in all mice (no hindlimb movement, BMS score at 0), with limited spontaneous recovery that plateaued at paresis (only isolated ankle movements, average BMS score at 1). None of these injured mice displayed any weight support or stepping for up to 20 weeks after injury (Fig. 2b).

To assess the effects of activating the neurons expressing hM3Dq, we treated the mice with clozapine-N-oxide (CNO), a ligand for DREADDs, every 2 weeks after injury (Fig. 2b). In all tested time points, mice with control virus had no improvements after CNO treatment, but the mice expressing activating hM3Dq exhibited significantly improved hindlimb locomotor function, i.e., hindlimb plantar placement or dorsal stepping with an average BMS score of 3 (Fig. 2b, Supplementary Video 1). At 1 h after CNO treatment, a majority of hM3Dq-expressing mice (92%) regained significant weight-bearing dorsal or plantar stepping capabilities, while a small portion (8%) only achieved plantar placement of the hindlimbs (Supplementary Fig. 3b). The extent of improved functional performance depended on the timing after CNO administration (Supplementary Fig. 3a, Supplementary Video 1), likely reflecting the clearance of CNO from circulation. Detailed hindlimb kinematics revealed the following major improvements: (1) a change from dragging flaccid hindlimbs to a standing pose (Fig. 2c); (2) increased weight support (increased iliac crest height, Fig. 2c, f); and (3) regained hindlimb stepping ability indicated by foot/toe lifting height (Fig. 2c, g), ankle range of motion (Supplementary Fig. 3g), and the amplitudes of hindlimb iliac crest height (Supplementary Fig. 3d). Importantly, the hindlimb of these treated mice exhibited similar cycle duration and stance/swing ratio as intact mice (Fig. 2d, e) and stepping frequency as their forelimb (Supplementary Fig. 3h). Although alternate stepping of hindlimbs was observed in these CNO-treated mice, de-couplings between steps during free walking prevented accurate quantification of interlimb coordination. Taken together, these results suggest that enhancing excitation of neurons in the spinal thoracic levels enabled the completely paralyzed mice to perform hindlimb stepping with body weight support.

On the other hand, although many neurons rostral to the lesion also expressed hM3Dq, we did not observe detectable behavioural differences in their forelimb upon CNO treatment (Supplementary Fig. 3i, j). Consistent with improved hindlimb stepping ability, EMG recordings indicate alternate firing of the ankle flexor tibialis anterior muscle (TA) and the extensor gastrocnemius soleus muscle (GS). On the other hand, the proximal joints and their muscles, such as knee extensor vastus lateralis (VL) and flexor ST muscle, were also recruited during walking, but with much lesser extent (Supplementary Fig. 4a, b). Together, our data are indicating that CNO-treated mice exhibited hindlimb movement that is mainly driven by the ankle joint. The movements of other proximal joints might be associated with passive dragging. Remarkably, such improvements were still triggered by CNO at 10 or 20 weeks after injury (Fig. 2b), suggesting the efficacy of manipulating the excitability of thoracic neurons in both acute and chronic SCI models.

At the end of the experiments, we verified the complete injury by immunohistochemistry with anti-5-HT antibody on transverse sections at T10 and L2 (Supplementary Fig. 5a, b). With this tail vein injection procedure, numerous mCherry+ axons, but not cell bodies, were seen in the lumbar spinal cord, consistent with the efficient labelling of descending-projecting propriospinal neurons (Supplementary Fig. 4c). Thus, these results suggest that upon CNO treatment, activation of T8-13 neurons could indirectly enhance the overall excitatibility of lumbar spinal cord through propriospinal circuits and facilitate hindlimb locomotion. In support of this, we found that hindlimb responses to noxious stimuli were partially recovered following CNO application (Supplementary Fig. 4d, e).

While these experiments involve the activation of thoracic neurons, we also examined the outcomes of inhibiting these neurons. As expected, injured mice expressing the inhibitory hM4Di-DREADD in thoracic neurons failed to improve behavioural outcomes upon the same CNO treatment (Supplementary Fig. 4f). Thus, these results suggest that activating the thoracic neurons, including descending propriospinal neurons, allows mice with nearly complete paralysis to achieve significant hindlimb locomotor function.

### Activating excitatory neurons in the thoracic spinal cord preferentially improves standing ability after T8 crush injury

**Fig. 3:**
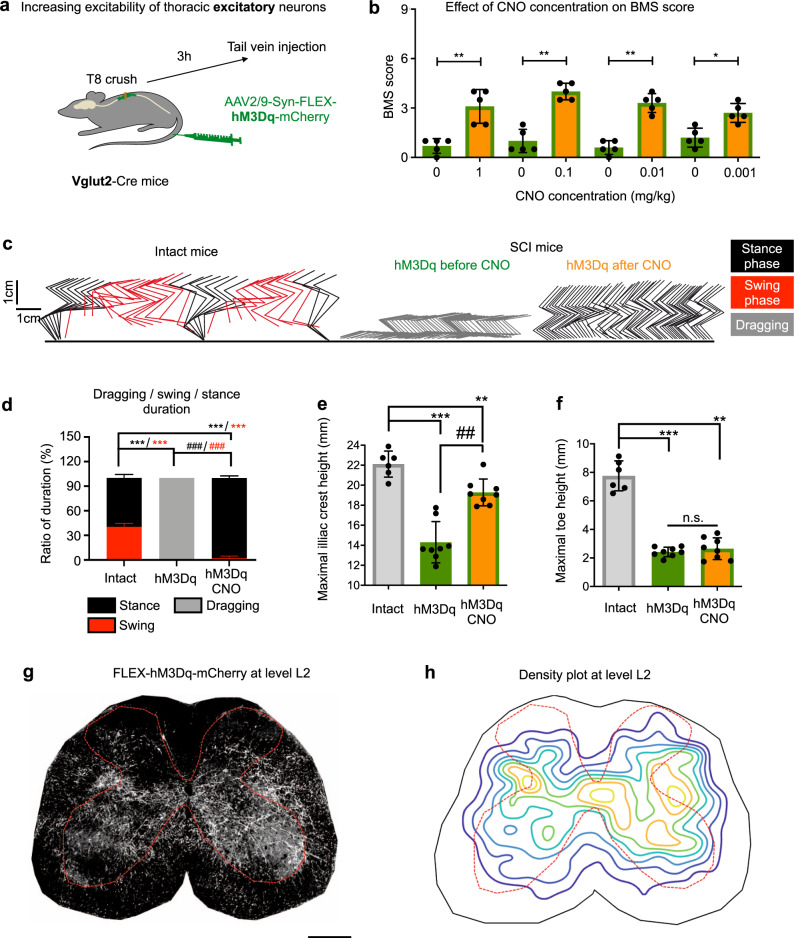
Activation of thoracic excitatory neurons improves standing but only minimal stepping in mice with T8 crush injury. **a** Experimental schematic. After a T8 crush injury, Vglut2-Cre mice received a tail vein injection of AAV2/9-Syn-FLEX-hM3Dq-mCherry and were then subjected to behavioural assessments at indicated time points. **b** BMS scores of Vglut2 mice expressing excitatory hM3Dq in thoracic neurons before and 1 h after CNO at different doses (*n* = 5). Data shown as mean ± SD (also for other panels in this figure). Two tailed paired *t* test; **p = 0.010 (1 mg/kg); 0.002 (0.1 mg/kg); 0.0013 (0.01 mg/kg); *p = 0.0132 (0.001 mg/kg). **c** Stickviews of kinematic analysis of intact mice and Vglut2 mice expressing excitatory hM3Dq before and after CNO (0.01 mg/kg). The phases of dragging, stance, or swing were marked in indicated colours. Data from intact mice are replotted from Fig. 2c for comparison. **d** Duration of swing, stance and dragging phases of different groups of mice with CNO (0.01 mg/kg). Data from intact mice are replotted from Fig. 2e for comparison. Two tailed sign test was used for within-group comparison (#) and Wilcoxon rank sum was used for between-group comparison (*). ***/^###^*p* < 0.001, *n* = 6 for each group. Quantification of the maximum iliac crest heights (**e**) and maximum toe heights (**f**). Two tailed paired *t* test for within group comparison (#) and 2 sample *t* test for between group comparison (*). **p = 0.0019; ^##^*p* = 0.0078, ***p < 0.001 and n.s. *p* = 0.5001. *n* = 6 (intact) and *n* = 8 (hM3Dq). Data from intact mice are replotted from Fig. 2f, g for comparison. A representative mCherry image (**g**) and average density plot (**h**) of an L2 transverse section showing the distribution of descending projection axons in the lumbar spinal cord of injured Vglut2-Cre mice with injection of AAV2/9-Syn-FLEX-hM3Dq-mCherry. *n* = 3 mice, 3–4 sections per mouse. Scale bar: 250 µm.

Since propriospinal neurons could physiologically act as either excitatory or inhibitory drivers^13,14,33^, we next assessed whether manipulating each of these populations separately might give rise to different behavioural outcomes. Thus, we first examined the effects of selective activation of the excitatory population by utilizing Vglut2-Cre transgenic mice, which express Cre in excitatory spinal neurons^34^. Vglut2-Cre mice received tail vein injections of AAV2/9-Syn-FLEX-hM3Dq-mCherry at 3 h after T8 crush injury and were then subjected to locomotor assessments before and after CNO treatment at 4 weeks after injury (Fig. 3a).

Before CNO treatment, the mice had paralysis or paresis (BMS score of 0–1). However, at all tested concentrations of CNO (0.001–1 mg/kg), the hind limbs of vGlut2-Cre mice adopted a flexed position where the plantar paw surface gently draged across the ground surface, with or without reflexive-like stepping, with BMS scores of 3 (Fig. 3b, Supplementary Video 2)^32^. Even after lower dose of CNO treatment (0.01 mg/kg), their hindlimbs, in contrast to their forelimbs, only occasionally stepped, and had predominant “stance” with rare swing phases (Fig. 3c, d), spasm-like rigid distal joints, and limited foot flexion (toe-dragging) (Fig. 3c and Supplementary Video 2). As a result, the hindlimb stepping frequency was substantially lower than that of their forelimbs, with a ratio of 1:6.7 (Supplementary Fig. 6a). Furthermore, these mice had significantly increased iliac crest heights (Fig. 3e). However, compared with intact mice, these mice showed limited toe/foot lifting and hindlimb joints rotation capability (Fig. 3f, Supplementary Fig. 6c–e), suggesting limited stepping improvements. It is noticeable that at higher doses of CNO (0.1–1 mg/kg) treated mice developed rapid and repetitive foot oscillations, likely signs of spasms (muscles jerks or repeated twitching) (Supplementary Fig. 6f and Supplementary Video 2). Importantly, similar results were also obtained in mice with delayed tail vein injections (1 day after injury) of AAV2/9-Syn-FLEX-hM3Dq-mCherry, though the effect was less pronounced (Supplementary Fig. 6g), further substantiating the effects of manipulating these thoracic neurons on hindlimb locomotor function.

In addition to verifying no spared serotonergic axons remained in the lumbar levels in mice with a T8 crush injury (Supplementary Fig. 5), histological analysis indicated that these descending-projecting mCherry+ neurons were mainly located in thoracic levels (T8-T12), but barely in the lumbar levels if at all (Supplementary Fig. 6h). At lumbar spinal cord levels, descending axons project widely, including around the dorso-lateral cord, the medial cord where most premotor neurons are located^13,35^, and ventral horn where the majority of motor neurons reside (Fig. 3g, h). Most striking is the bundle in the dorso-lateral spinal cord, reminiscent of the “stepping strip” described by Kazennikov et al. in cats^36^. Thus, these excitatory descending projections may directly or indirectly engage motor neurons. Taken together, our results show that recruiting excitatory neurons in the thoracic levels leads to the recovery of significant weight-bearing capacity but limited stepping ability.

### Inhibiting inhibitory neurons in the thoracic spinal cord preferentially promotes stepping in mice with T8 crush injury

**Fig. 4:**
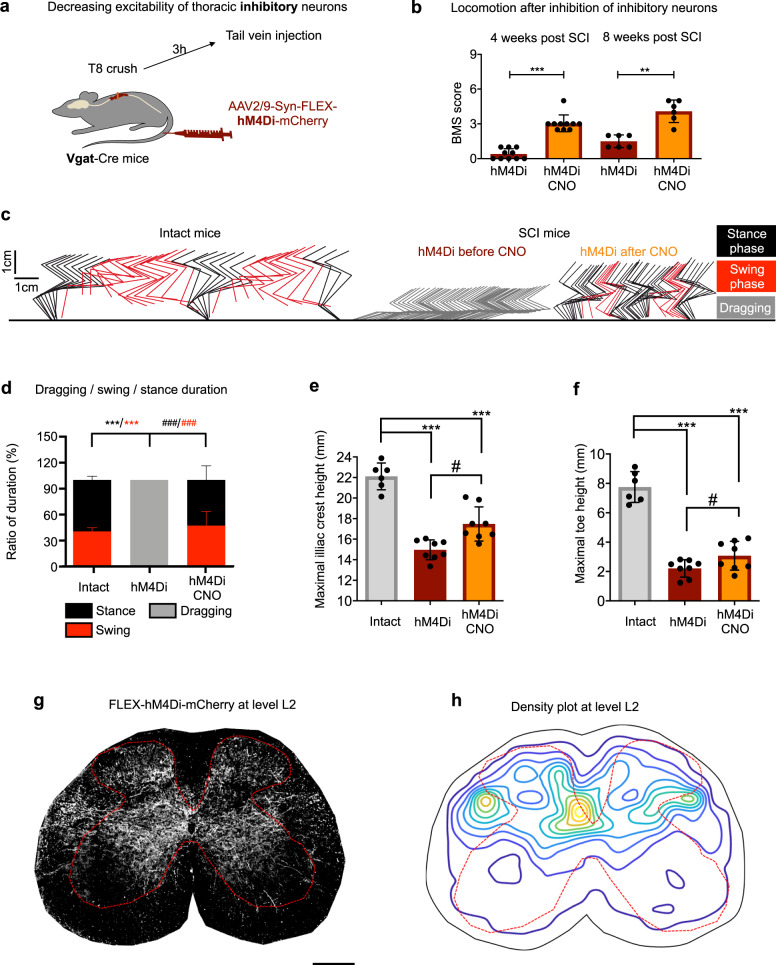
Inhibition of thoracic inhibitory neurons preferentially improve stepping ability in mice after T8 crush. **a** Experimental schematic. After a T8 crush injury, Vgat-Cre mice received a tail vein injection of AAV2/9-Syn-FLEX-hM4Di-mCherry. **b** BMS scores of Vgat-Cre mice with hM4Di expression in thoracic neurons before and 1 h after the application of 5 mg/kg CNO at 4 and 8 weeks past SCI. Data shown as mean ± SD (also for other panels in this figure). Two tailed paired *t* test. ***p < 0.0001 (4 weeks); **p = 0.0020 (8 weeks). *n* = 10 (4 weeks) and *n* = 6 (8 weeks). **c** Stickviews of detailed kinematic analysis in intact mice and Vgat mice with hM4Di expression in thoracic neurons before and 1 h after CNO (5 mg/kg). The phases of dragging, stance, or swing were marked in indicated colours. Data from intact mice are replotted from Fig. 2c for comparison. **d** Duration of swing, stance, and dragging phases of different groups of mice. Data from intact mice are replotted from Fig. 2e for comparison. Two tailed sign test was used for within-group comparison (#) and Wilcoxon rank sum was used for between-group comparison (*) *n* = 6 (intact group) and *n* = 8 (SCI group). ***p < 0.001. Quantification of maximum iliac crest heights (**e**) and maximum toe heights (**f**). Two tailed paired *t* test for within-group comparison (#) and 2 sample ttest for between-group comparison (*). ^#^*p* = 0.0045 (**e**); 0.0191 (**f**) and ***p < 0.0001. *n* = 6 (intact) and *n* = 8 (hM4Di). Data from intact mice are replotted from Fig. 2f, g for comparison. A representative mCherry image (**g**) and average density plot (**h**) of an L2 transverse section showing the distribution of descending projection axons in the lumbar spinal cord in injured Vgat-Cre mice with a tail vein injection of AAV2/9-Syn-FLEX-ChR2-mCherry. *n* = 3 mice, 3–4 sections per mouse. Scale bar: 250 µm.

Next, we tested if silencing inhibitory neurons in thoracic levels would mirror the effects seen after activating excitatory neurons. To do this, Vgat-Cre transgenic mice, labelling inhibitory neurons^34^, received tail vein injection of AAV2/9-Syn-FLEX-hM4Di-mCherry at 3 h after complete spinal cord crush (Fig. 4a). Since CNO has a low binding affinity for hM4Di, higher CNO concentrations than for hM3Dq experiments were administrated. Upon CNO treatment with 5 mg/kg at either 4 or 8 weeks after injury, the majority of mice achieved hindlimb stepping ability with an average BMS score of 3 (Fig. 4b–d, Supplementary Fig. 7a, Supplementary Video 3). Consistently, these mice had improved toe lifting and hindlimb joints (especially ankle joint) rotation (Fig. 4f, and Supplementary Fig. 7c–e), and iliac crest height amplitude (Supplementary Fig. 7b), to an extent similar to that seen in intact mice. However, the same mice displayed only mild improvement in body weight support (Fig. 4e), consistent with the observations that they largely failed to elevate their trunks or prevent knee striking with the ground (Supplementary Video 3). On the other hand, in contrast to the limb/muscle spasms observed with activation of excitatory neurons at higher CNO concentrations, no spastic events were observed in these mice with all doses of CNO tested (up to 10 mg/kg).

As expected, we did not observe any spared axons in the lumbar level of these crushed mice (Supplementary Fig. 5). Additional histological analysis indicated that in contrast to excitatory propriospinal neurons, axons from the inhibitory descending-projecting neurons mainly distribute in the intermediate and dorsal spinal cord close to the central canal (Fig. 4g, h). Interestingly, the territory of such inhibitory neurons are largely complementary to those of excitatory propriospinal axons, i.e., avoiding the ventral zone or the entire dorsal horn (compare to Fig. 3g, h). Taken together, these results suggest that excitatory and inhibitory propriospinal neurons project to different regions of the lumbar spinal cord and thus impact different aspects of hindlimb locomotion. Specifically, while silencing inhibitory neurons mainly improves hindlimb stepping frequency, primarily due to ankle flexion, activating excitatory projecting propriospinal neurons leads to spasm-like episodes (muscle spasms) with weight-bearing plantar foot placement but limited stepping.

### Activation of lumbar-projecting thoracic propriospinal neurons is sufficient for eliciting locomotor behavioural after injury

**Fig. 5:**
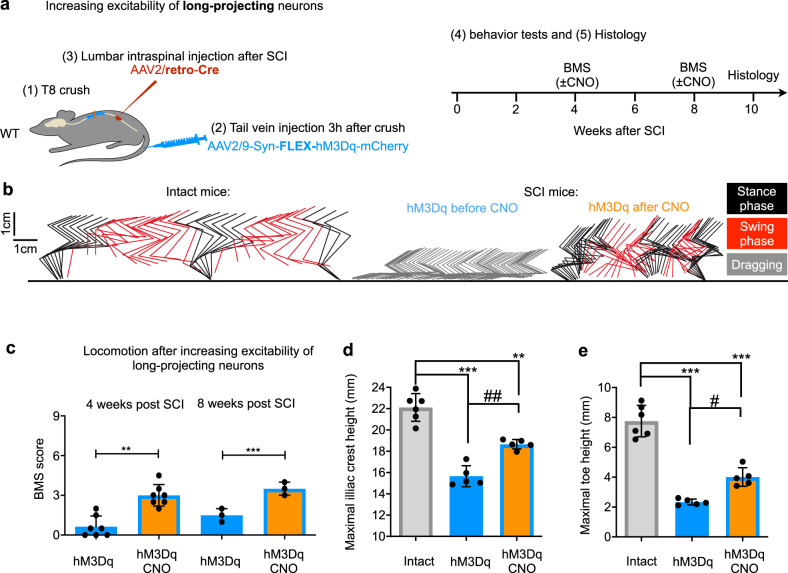
Activation of propriospinal neurons that project from thoracic to lumbar levels elicits locomotor behavioural recovery after T8 crush. **a** Experimental schematic of the intersectional strategy of limiting hM3Dq expression to propriospinal neurons projecting to lumbar spinal cord. **b** Stickviews of kinematic analysis of intact mice and injured mice with targeted expression of hM3Dq in long-projecting neurons before and during CNO (1 mg/kg). The phases of dragging, stance, or swing were marked in indicated colours. Data from intact mice are replotted from Fig. 2c for comparison. **c** BMS scores of mice with targeted expression of hM3Dq in long-projecting neurons at 4 and 8 weeks after injury, before and 1 h after application of 1 mg/kg CNO. Data shown as mean ± SD. Two tailed paired *t* test. **p = 0.0002 (4 weeks) ***p < 0.0001 (8 weeks). *n* = 7 (4 weeks) and *n* = 3 (8 weeks). Quantification of the maximum iliac crest heights (**d**) and the maximum toe height (**e**). *n* = 6 (intact) and *n* = 5 (hM3Dq). Data from intact mice are replotted from Fig. 2e, f for comparison. Shown are means ± SD. Two tailed paired *t* test for within-group comparison (#) and two sample *t* test for between-group comparison (*). ^##^*p* = 0.0030, **p = 0.0003, ***p < 0.001 and ^#^*p* = 0.0017.

The experiments described above relied on non-selective manipulation of thoracic neurons, including propriospinal neurons projecting to the lumbar spinal cord and other spinal segments. We next examined whether selective manipulation of propriospinal neurons that project axons to the lumbar cord is sufficient to mediate functional performance. To do this, we modified our experimental procedure by injecting retro-AAV-Cre into the lumbar spinal cord 7–10 days after T8 crush and tail vein injection of AAV2/9-Syn-FLEX-hM3Dq-mCherry 3 hr after injury (Fig. 5a). As retro-AAVs can efficiently transduce neurons via their axonal terminals^37^, this intersectional procedure led to the expression of hM3Dq-mCherry in thoracic propriospinal neurons that project into the lumbar cord (Supplementary Fig. 8). Before CNO application, all mice were paralyzed with an average BMS score below 1. However, CNO treatment led to significant behavioural improvements (with an average BMS score of 3) (Fig. 5b, c), to extents similar to non-selective hM3Dq-mCherry expression in wild-type mice (Fig. 2). Mice displayed significant improvements in body weight support, as shown by increased heights of the iliac crest and hip joints after CNO treatment (Fig. 5d), as well as improved stepping ability as indicated by increased toe height (Fig. 5e).

### Manipulating neurons in lumbar spinal cord triggers severe spasms without locomotor function after T8 complete crush injury

The experiments above demonstrate that manipulating thoracic propriospinal neurons impacted neuronal circuits in the lumbar spinal cord for functional improvement, presumably by indirectly driving local lumbar circuits like the CPG or lumbar motor pools. We next asked if directly manipulating neurons in the lumbar spinal cord might have similar behavioural outcomes. For this, we optimized a protocol for stereotaxic injection of AAVs to the lumbar spinal cord (Supplementary Fig. 9a), which led to mCherry expression across most of the lumbar cord (Supplementary Fig. 9b). In order to probe the contribution of different neuronal populations, we injected AAV2/1-Syn-hM3Dq-mCherry to the lumbar cord of wild-type mice (for activating all neurons), AAV2/1-Syn-FLEX-hM3Dq-mCherry to that of Vglut2-Cre mice (for activating excitatory neurons), and AAV2/1-Syn-FLEX-hM4Di-mCherry to that of Vgat-Cre mice (for inhibiting inhibitory neurons) (Supplementary Fig. 9a). After lumbar injection, overground locomotor behaviour was monitored for 7–10 days. Only mice showing no observable motor deficits were used for T8 crush injury and behavioural assessments. When tested with CNO treatment in the weeks to months post-injury, no meaningful functional locomotion was observed in any group. Instead, all groups exhibited strong and frequent muscle spasms, albeit with different patterns (Supplementary Fig. 9c, d Supplementary Video 4). By using the Penn spasm frequency scale^38^, we found that all mice reached a score of 4 out of 4, with more than 10 spontaneous spasms/hour (Supplementary Fig. 9e).

Interestingly, different patterns of muscle spasms were observed in each group (Supplementary Fig. 9c). Lumbar expression of hM3Dq in wild-type mice led to spasms of the legs, most pronounced in hip flexors (likely gluteus medius, Supplementary Fig. 9d), with an overall stiffness and forward/inward stretching of the hindlimbs. This phenotype was so pronounced that mice almost always fell towards one side of their body and dragged their extended hindlimbs on the other. Selective expression of hM3Dq in Vglut2-Cre mice led to similar observations, but these mice had episodes of random spasms. In general, these mice often stood still, seemingly unwilling to walk, during which no leg spasms were observed. However, when mice were lifted up during such periods, they showed tremor-like shaking of their paws with spontaneous bursts of leg spasms. On the other hand, in all Vgat-Cre mice with hM4Di expression, we observed extension of the hindlimbs behind the body and continuous bilateral spasms.

EMG recordings revealed random firing of all muscle groups tested, but with some differences: (1) in both wild-type and Vglut2-Cre mice with hM3Dq expression, strong activation of hip extensors was observed, and (2) in Vgat-Cre mice with hM4Di expression, non-stopping co-activation of ankle extensor and flexor muscles occurred (Supplementary Fig. 9d, f). Such different forms of spasms were unlikely due to the variation in surgical injection, as the same procedure was used in all mice (one example of viral mediated tdTomato expression in lumbar spinal cord is shown in Supplementary Fig. 9b), and relatively consistent behaviour was observed in all mice of each group. Together, our results suggest that direct manipulation of lumbar spinal neurons failed to lead to patterned activation with meaningful behavioural improvements.

## Discussion

In this study, we optimized a non-invasive intravenous injection method that can efficiently target neurons, and other cell types, extending at and adjacent to a spinal cord lesion over a clinically relevant time frame. With this procedure, we introduced chemogenetic DREADDs to thoracic neurons in mice with a complete thoracic SCI and demonstrated that modulating their excitability triggered consistent and significant hindlimb stepping, a movement that primarily recruits ankle joint flexion and extension. Intriguingly, distinct behavioural outcomes were observed after altering excitatory or inhibitory propriospinal neuron activity in the thoracic spinal cord, with preferential improvements on standing when activating excitatory neurons or stepping when inhibiting inhibitory neurons.

### Efficient AAV delivery to the SCI epicentre

Neurons and glial cells around the SCI epicentre are most affected and should be a primary target of therapeutic interventions. However, there is no efficient method of targeting these cells efficiently and selectively. Taking advantage of transiently compromised BSCB after injury, we demonstrated that AAV2/9 vectors, if injected within three days after SCI, are able to transduce the majority of cells around the lesion site. Interestingly, not all serotypes of AAV vectors could do the same, as some, such as AAV2/5 and AAV2/6, transduced few cells even with similar numbers of viral particles injected. Thus, it is unlikely that the blood spinal cord barrier is totally opened to allow all types of AAVs to enter the spinal cord even after injury. Such variation in AAV serotype penetration might be relevant to their binding properties with cells in the BSCB. In particular, AAV2/9 was shown to possess some capacity of crossing the blood brain barrier (BBB), most noticeably in neonatal ages^39^. Alternatively, some serotypes could have tropisms for cells in other organs, which would reduce their likelihood of binding to cells of the BBB.

Within the CNS, variance in cellular tropism between different AAV serotypes has been observed after direct injections^40,41^ or systemic delivery^42^. Surprisingly, we did not see major differences in cellular tropism within the spinal cord after BSCB mediated crossing of any tested AAV serotypes. This might be due to the unique delivery method. It is also possible, that the use of H2B-GFP in our experiments allowed for more sensitive and unambiguous detection of positive cells. Indeed, the distribution of cellular tropism among most of our serotypes was similar to those reported for PHP.B after tail vein injection in which H2B-GFP was used as a reporter^43^.

To specifically deliver target genes to CNS cell types whose infection is desired, one strategy is to use promoters specific for these cells, like glial fibrillary acidic protein promotor for limiting expression to astrocytes or synapsin promotor for exclusive expression in neurons. This makes intravenous injection of AAVs after SCI a convenient method to express desired genes broadly throughout the injury site while preventing gene expression in non-injured CNS areas or non-CNS organs.

### Propriospinal pathways as a target for promoting functional recovery

For function restoration in both animal models and human patients with SCI, a key question is how to activate the intrinsic neuronal circuits within the spinal cord. In SCI patients electrical stimulation has been used successfully to restore locomotor function^3,4,44^. Despite these exciting clinical successes, the mechanisms underlying such electrical neuromodulation are still under debate^16,45^.

Currently, proposed targets include axons in the dorsal roots, longitudinal spinal fibres of the posterior column, and propriospinal networks, among others. Because of a lack of direct assessments of these neuronal circuits, most efforts in this direction have relied on computational methods^46,47^. In assessing the behavioural outcomes when manipulating different cohorts of neurons, our results revealed strikingly diverse outcomes between lumbar-projecting thoracic proprospinal neurons (remote control) and local neuronal networks in the lumbar spinal cord (direct control). Engagement of such remote control resulted in advantageous patterned activity with functional stepping recovery, while direct control lead to unwanted hindlimb spasms. Thus, our results point to these neurons as potentially powerful targets for activating lumbar CPGs and triggering behaviourally relevant activity patterns. In addition to assessing the engagement of these long-projecting propriospinal pathways by electrical stimulation, future studies should optimize such protocols to recruit different remote control pathways for maximizing functional recovery.

### Unique projection patterns and function of excitatory and inhibitory propriospinal projections

Maintaining precise excitatory/inhibitory electrochemical balance within neuronal networks is essential for all types of behaviour. This is largely achieved by dynamic activation of excitatory and inhibitory neurons. Interestingly, we found that activating excitatory propriospinal neurons allowed paralyzed mice to stand, but inhibiting inhibitory neurons preferentially promotes the recovery of stepping capacity. Furthermore, their descending axons project and terminate in different yet complementary regions of the lumbar spinal cord. Excitatory axons terminate in most spinal areas (excluding dorsal horn) with two prominent concentrations in ventro-medial and dorso-lateral spinal cord, while inhibitory axons are seen around the central canal, in particular the dorso-medial regions. Because spinal motor neurons are located in the ventral spinal cord, it is conceivable that excitatory inputs recruit motor neurons directly and their pre-motor neurons for coordinated muscle activation (muscle strength). On the other hand, the dorso-medial spinal cord hosts many commissural neurons with their projections to their contralateral cord^48^. Thus, inhibitory propriospinal inputs may be more suited for bilateral coordination required for stepping. This may also explain the spasms associated with activation of excitatory propriospinal neurons but not inhibition of inhibitory neurons.

The striking axonal bundles from the excitatory descending axons were observed in the dorso-lateral funiculus, which might be relevant to the “stepping strip” described by Kazennikov et al.^34^. In their studies, they showed that in mesencephalic cats micro-stimulation of the dorso-lateral funiculus is sufficient to elicit hindlimb stepping^36,49^. In addition, Chung et al. also reported that many propriospinal axons project into the dorsal funiculi or dorso-lateral funiculus^49–51^. It would be interesting (meaningful) to discern the physiological function and translational potential of such longitudinal projections.

Intriguingly, previous studies showed that standing or stepping could be improved in task-specific manner in spinally transected cat^52–54^. When trained specifically to stand, these cats could stand but not walk. On the other hand, when these animals were trained to walk, they could not stand^52,53^. However, relevant neural substrates for supporting standing or stepping remain unknown. Based on our results, it would be interesting to assess whether task-specific training may preferentially recruit excitatory or inhibitory propriospinal neurons and if training and our manipulations could be combined for better rehabilitative outcomes.

In conclusion, in a complete thoracic SCI model, we demonstrated the feasibility of achieving functional locomotion by manipulating propriospinal neurons in the thoracic levels. Future studies will investigate whether this approach could be combined with regenerative strategies such as axon regeneration^55–57^ and stem cell grafting^58^ to enhance both automatic and intentional functional recovery in the conditions of complete or incomplete SCI. As AAV2/9 is increasingly used for gene therapy^59,60^, it is conceivable that technology like DREADD-assisted neuronal mondulation could be useful for functional restoration in clinical settings.

## Methods

### Mouse strains

Wild-type C57BL/6 mice were purchased from Charles River Laboratories. Vglut2-Cre and Vgat-Cre mice were obtained from Jackson Laboratory (Jax stock # 016963 and 028862, respectively). Surgeries were conducted on adult female mice (18–21 g) at the age of 8–10 weeks. All experiments were performed in compliance with protocols approved by the IACUC at Boston Children’s Hospital. Mice were given ad libitum access to food and water, and housed in cages under 12 h day/night cycles with bedding changed frequently. Mice were not permitted to breed before or during their inclusion in in vivo experiments.

### Plasmids and viruses

pAAV-Syn-GFP was a gift from Edward Boyden (Addgene plasmid # 58867), AAV-FLEX-ChR2-tdtomato was a gift from Scott Sternson^61^ (Addgene plasmid # 18917), pAAV-hSyn-hChR2(H134R)-mCherry was a gift from Karl Deisseroth (Addgene plasmid # 26976), and pAAV-hSyn-DIO-hM3D(Gq)-mCherry, pAAV-hSyn-DIO-hM4D(Gi)-mCherry, and pAAV-hSyn-DIO-mCherry were a gift from Bryan Roth^62^ (Addgene plasmid # 44361, 44362 and 50459). pAAV-CAG-FLEX-H2B-GFP was purchased from Vigenbio. pAAV-Syn-mCherry and pAAV-Cre was constructed as described earlier^22,63^. pAAV-CAG-H2B-GFP was constructed by replacing GFP in pAAV-CAG-GFP (developed by Z. He lab) with H2B-GFP (from Addgene plasmid # 11680). All plasmids were packaged into AAVs by the Boston Children’s Hospital Viral Core. These AAV vectors include: AAV-2/1, 2, 2/5, 2/6, 2/7, 2/8, 2/9, 2/10, retro-AAV, and DJ-CAG-H2B-GFP, AAV2/9-Syn-H2B-GFP, AAV2/9-CAG-FLEX-H2B-GFP, AAV2/9-Syn-hM3Dq-mCherry, AAV2/9-Syn-ChR2-tdTomato, AAV2/9-Syn-FLEX-hM3Dq-mCherry, AAV2/9-Syn-FLEX-hM4Di-mCherry, Retro-AAV-Cre, AAV2/1-Syn-hM3Dq-mCherry, AAV2/1-Syn-FLEX-hM3Dq-mCherry, and AAV2/1-Syn-FLEX-hM4Di-mCherry.

### Surgeries

#### T8 crush injury

The procedure is similar to what described previously^26,27^. Mice received pain medication (buprenorphine, 0.05 mg/kg) 1 h before surgery and were anaesthetised with ketamine/xylazine (100/10 mg/kg). To prevent xerophthalmia during anaesthesia, both eyes were covered with eye lube. The back fur was shaved, the skin overlying the vertebral column was incised, and the muscles were detached from the vertebra. A single-level bilaminectomy (complete removal of the dorsal arch of the vertebrae: processus spinosus and bilateral lamina arcus vertebrae) was then performed to expose the spinal cord at the level of T8. After opening the dura mater, the spinal cord was crushed with fine forceps (Fine Science Tools), resulting in a complete injury. The wound was rinsed with normal saline and closed in layers. After SCI, 1 ml of saline was injected subcutaneously. Mice were kept in 25 °C incubator for less than 48 h. Postoperative care comprised of analgesic treatment for three days following SCI and manual bladder compression twice daily. Any urinary infection was treated with an antibiotic therapy (Baytril, 10 mg/kg, for 7 days). The body weight of each mouse was measured weekly and any presenting more than a 15% decrease in body weight was sacrificed.

#### Tail vein injection

For tail vein injections mice were anesthetized with isoflurane (1.5–2% in 2:3 O_2_/N_2_O, inhaled). The tail was washed with 70% ethanol and 200 µL virus diluted in saline were injected into the tail vein through a syringe with a 30 G needle. For serotype analysis (Figs. 1A and S1), we used 8•10^12 gc per mouse (200 µL of 4•10^13 gc/mL) of virus titered with ITR primer (Supplementary Table 1). For AAV2 where such high titre can not be produced, we used the highest reasonably available titre of 8•10^11 gc per mouse. For functional studies, we used virus titered with WPRE. Since these primers generally result in an approximately 40-fold lower titre, we injected 200 µL of 1*10^12 gc/mL, resulting in 2•10^11 gc/mouse.

#### Lumbar spinal cord injection

For retrograde tracing experiments, lumbar spinal cord injection was conducted 7–10 days after both the T8 crush and subsequent tail vein injection. As described previously^22,64^, retro-AAV was injected into left and right lumbar spinal cord from segments L2-L4. Injection coordinates were 0.5 mm lateral of the midline. 3 injection sites were separated 1 mm away, and at each site 2 injections were performed at 2 different depths, 0.6 mm and 1.2 mm (in total 6 injections per side, 100 nl per injection). This procedure was also used for the experiments in Fig. 5 where AAV vectors were injected into the lumbar spinal cord 7–10 days before T8 crush.

#### CNO injection

In mice that received DREADD-expressing AAVs, injections of clozapine-N-oxide (CNO, BML-NS105, Enzo Life Sciences Inc) were given i.p. at a concentration of 1 mg/kg for hM3Dq (0.001–1 mg/kg in Vglut2 mice as indicated in Figs. 3) and 5 mg/kg for hM4Di and control virus, at indicated time points.

### Histology

Anesthetized mice were transcardially perfused with 4% paraformaldehyde (PFA). Dissected spinal cords were post-fixed in 4% PFA overnight, and then immersed in 30% sucrose solutions at least two days before embedding and snap-freezing in OCT. 20 µm thick sections were cut for cross sections and taken up on coated glass slides and 40 µm sections were cut for free floating and kept in PBS at 4 °C. For serial sections, the spinal cord segments containing the lesion (T6 to L5) were cut in the horizontal plane. Serial sections (40 μm) were collected spanning the ventral to dorsal axis. Every four sections were stained for 5-HT + axons and the number quantified. For immunohistochemistry, tissue was blocked for one hour in PBS with 5% donkey serum and 0.3% Triton X-100, and then incubated overnight at 4 °C in primary antibodies, diluted in PBS with 5% donkey serum and 0.3% Triton X-100, followed by treatment with secondary antibodies (Jackson ImmunoResearch Laboratories Inc. or Invitrogen) for 1–2 h at room temperature after washing 3 times for 5 min each. Primary antibodies used were: chicken anti-GFP (1:1000, Abcam ab13970); rabbit anti-RFP (1:500, Abcam ab34771); mouse anti-NeuN (1:500, Millipore MAB377); rabbit anti-GFAP (1:500, Dako Z0334); rabbit anti-Olig2 (1:300, Millipore AB9610); rabbit anti-PDGFRβ (1:500, Thermo Fisher Scientific MA5-15143); rat anti-CD68 (1:500, BioRad MCA1957); rat anti-CD31 (1:1:500, BD Biosciences 550274); and goat anti-5-HT (1:200, Immunostar 20079). The following secondary antibodies were used at 1:400 final dilution: 488 Donkey anti-Chicken (703-545-155), 488 Donkey anti-Rabbit (711-545-152), Biotin Donkey anti-Rabbit (711-066-152), HRP Donkey anti-Rabbit (715-035-151), Biotin Donkey anti-Rat (712-065-153), HRP Donkey anti-Mouse (712-035-153), Biotin Donkey anti-Mouse (715-065-151), HRP Donkey anti-Mouse (715-035-150) from Jackson ImmunoResearch, 568 Donkey anti-Rabbit (A10042) and 647 Donkey anti-Goat (A-21447) from Thermo Fisher Scientific (Invitrogen) and 647 Streptavidin (S-21374) and 568 Streptavidin (S-11226) from Life Technologies. NeuN, CD31 and PDGFRβ signals were amplified with a tyramide amplification (TSA Cyanine 5 Kit, Perkin Elmer SAT705A001EA). Olig2 and CD68 signals were amplified with a biotin/streptavidin step between primary and secondary with biotinylated antibodies raised against the primary antibody’s host species. For 5-HT stainings, Triton-X100 was raised to 2% during the primary and washing steps, and primary incubation was performed at room temperature. After staining, tissue was washed, incubated with DAPI in the last wash step, adhered to room-temperature charged microscope slides, and mounted with VECTASHIELD Antifade Mounting Medium (Vector Laboratories, H-1200).

### Microscopy and semi-automated cell counting

Fluorescent images were acquired using a Zeiss LSM710 Confocal Microscope in the IDDRC Imaging Core of Boston Children’s Hospital. Brightness and contrast of the images were adjusted, pseudo-colours applied for presentation and images cropped with imageJ (NIH). When imaging was taken for quantification, image capture and processing were kept constant. 4–5 slides across the dorsal-ventral axis were imaged per spinal cord.

A home-developed Matlab (MathWorks, Inc) algorithm was used for cell quantification. Circle-like cells were identified using Circular Hough Transform method. Cell numbers and coordinates in each channel were exported. Then virus efficiencies were quantified by counting the colocalized signals in different channels.

Figure 1b background of images were removed using rolling ball background subtraction method. Then in each image, a segmented line was drawn to cover the spinal cord area. The intensity values along the segmented line direction were exported. Intensity values were aligned according to the injury location and averaged in each serotype.

Figures 3h and 4h images were converted to binary images using a predefined threshold.

For each section, three to four images were acquired from each sample, which was repeated across a total of 3 animals and used to create the contour plots. All images were resized to equal dimensions and a meshgrid was applied to analyse the number of bright pixels within each square. The density values of each image were normalized and averaged. A contour plot was drawn to illustrate the axon density in each section.

### Behavioural assessments

Overground motor function was evaluated with a locomotor open field rating scale on the BMS^32^. fter baseline testing, mice received systematic administration (i.p.) of CNO. At 1–5 h after CNO injection, BMS scoring was re-examined in the same set of mice.

For detailed kinematic analysis, mice were placed in a clear plexiglass runway (80 cm long, 4 cm wide, and 12 cm high) to assess overground locomotion. Anatomical landmarks at the iliac crest, hip joint, knee joint, ankle, last toe, and L1 and L4 vertebrate were highlighted. For each mouse, at least 6 continuous gait cycles were captured by iPhone with 60 fps. During video acquisition, we focused on the central area (40 cm) of the plexiglass runway, where each mouse either walks, drags, and/or steps continuously for about 6–10 gaits. However, we cannot rule out errors as a result of quickly moving mice and modest speed of the iPhone camera. If the hindlimbs did not move, we used the forelimbs to determine the gait. For each mouse, we recorded at least 3 trials. The videos were chosen for further analysis based on the following criteria: (1) Continuous movement in the recording region; (2). Forward movement without head rotation. These videos were analysed by using DeepLabCut^65^ to trace the joints automatically. The kinematics were then analysed blindly and a stick view of hindlimb movements was drawn by MATLAB. Different individuals collected behavioural data and performed kinematic analyses. For the supplementary videos, mice without highlighted anatomical landmarks moved freely along the edge of a table while videos were recorded from a side view.

For analysing gait cycle, including angular excursions, oscillation of hip, knee, and ankle, and the horizontal hindlimb excursions, we followed the procedure described in Alluin et al.^66,67^, and Zorner et al.^68^. Intralimb coordination was analysed with the method described in Courtine et al.^20,63^.

For quantification of spasms of Vglut2 mice (Fig. 3), mice were placed on a transparent surface and videotaped from below for 3 h after CNO application. Spastic episodes were manually counted over a 5 min period at 60 min post CNO treatment. To quantify spastic events after lumbar injection (Supplementary Fig. 9e), mice were observed on an open surface and assessed using a mean spasm-frequency score ranging from 0 (on spasms on most days) to 4 (>10 spontaneous spasms/hour)^38^.

For noxious sensory behaviour tests, mice were first habituated to chambers (7.5 × 7.5 × 15 cm) for 60 min on 3 consecutive days. On the third day, animals were tested before and after CNO application (1, 2, 3, 4, 7, and 24 h) for their response to the following stimuli: pinprick or laser heat. For *pinprick*, a 30 G needle was applied ten times to the plantar surface of the hindlimb paw. A positive response was measured as stimuli-induced hindlimb movement including flinching, guarding, or jumping behaviour, which was often observed in parallel with spastic activity. For *laser heat*, a 447 nm laser beam, up to 200 mW, was also applied to the plantar surface of the hindlimb paw and the average time to paw withdrawal was calculated from three consecutive trials. Animals were returned to their home cage between measurements at 7 and 24 h.

### EMG recording

At indicated time points after T8 crush surgery, anesthetized mice underwent implantation of home-made bipolar electrodes into selected hindlimb muscles. Stainless steel electrodes (793200, A-M Systems Inc.) were led by 30 gauge needles and inserted into the mid-belly of gluteus maximus (GM), vastus lateralis (VL), semitendinosus (ST), tibialis anterior (TA), and lateral gastrocnemius (GS) of one hindlimb. A common ground wire was positioned subcutaneously in the neck-shoulder area. Wires were routed subcutaneously through the back to a connector securely cemented to the skull. Mice were allowed 7 days of recovery before subjected to behavioural assessment and EMG recording. EMG signals were acquired using differential AC amplifier (1700, A-M Systems Inc.) with 10–1000 Hz band-width filters, sampled at 4 kHz using a digitizer (PowerLab 16/35, ADInstruments Inc.), and analysed using LabChart 8 (ADInstruments Inc.). Acquired EMG signals and video recordings were synchronized using a digital light signal captured in both and to which the timeline was zeroed. CNO was administrated (i.p.) after 10 to 15 min of stable baseline recording. To record EMG signals during ground walking, mice were allowed to walk freely in an enclosed straight plexiglass runway while recording. To quantify EMG signals during spastic episodes, signals were rectified where 20-s episodes without obvious electromagnetic interference or movement artifacts from either baseline or 15 min after CNO were selected, and the area under curve (AUC) was calculated by integrating data points with sample interval. EMG recordings after CNO were normalized to the average baseline to eliminate variations from individual muscles.

### Quantification, statistics and reproducibility

Prior to statistical analysis, data were analysed by ESD (extreme studentized deviate) test. Assumptions (normality, equal variance) were tested by SAS 9.4 before applying parametric analyses. Paired, two-tailed Student’s *t* test or sign test was performed for comparison of the same group of mice before and after CNO treatment according to normality test result. Two sample *t* test or Wilcoxon rank sum was used for between group pairwise comparisons according to normality test result. The rest of the data were analysed using one-way or two-way ANOVA with or without repeated measures depending on the appropriated design. Multiple comparison procedures were carried out to identify specific between-group differences using Bonferroni’s post hoc. Error bars in all figures represent mean ± SD. Differences were considered statistically significant at *p* value below 0.05. *, **, ***, *p* < 0.05, 0.01, 0.001, respectively. All data were analysed using SAS 9.4 and GraphPad Prism 8.0. In all images the number of mice used is specified in the figure legend.

### Reporting summary

Further information on research design is available in the Nature Research Reporting Summary linked to this article.

## Supplementary information

Supplementary information

Supplementary Video 1

Supplementary Video 2

Supplementary Video 3

Supplementary Video 4

Description of additional supplementary files

Reporting Summary

Source code

## Data Availability

The data that support the findings of this study are available from the corresponding author upon reasonable request. Source data are provided with this paper.

## Source data

Source Data

## Code availability

All custom codes are provided with this paper.
