# Supplementary material for: Improving hindlimb locomotor function by Non-invasive AAV-mediated manipulations of propriospinal neurons in mice with complete spinal cord injury
Source: Nat Commun. 2021 Feb 3;12:781. doi: 10.1038/s41467-021-20980-4 (PMC7859413; doi:10.1038/s41467-021-20980-4)
Supplement: Supplementary file 1 — Supplementary information [file 41467_2021_20980_MOESM1_ESM.pdf]

SUPPLEMENTARY INFORMATION

"Improving Hindlimb Locomotor Function by Non-invasive AAV-mediated Manipulations of Propriospinal Neurons in Mice with Complete Spinal Cord Injury" by Brommer B., He M. et al., Nature Communications 2021.

Supplementary Figures

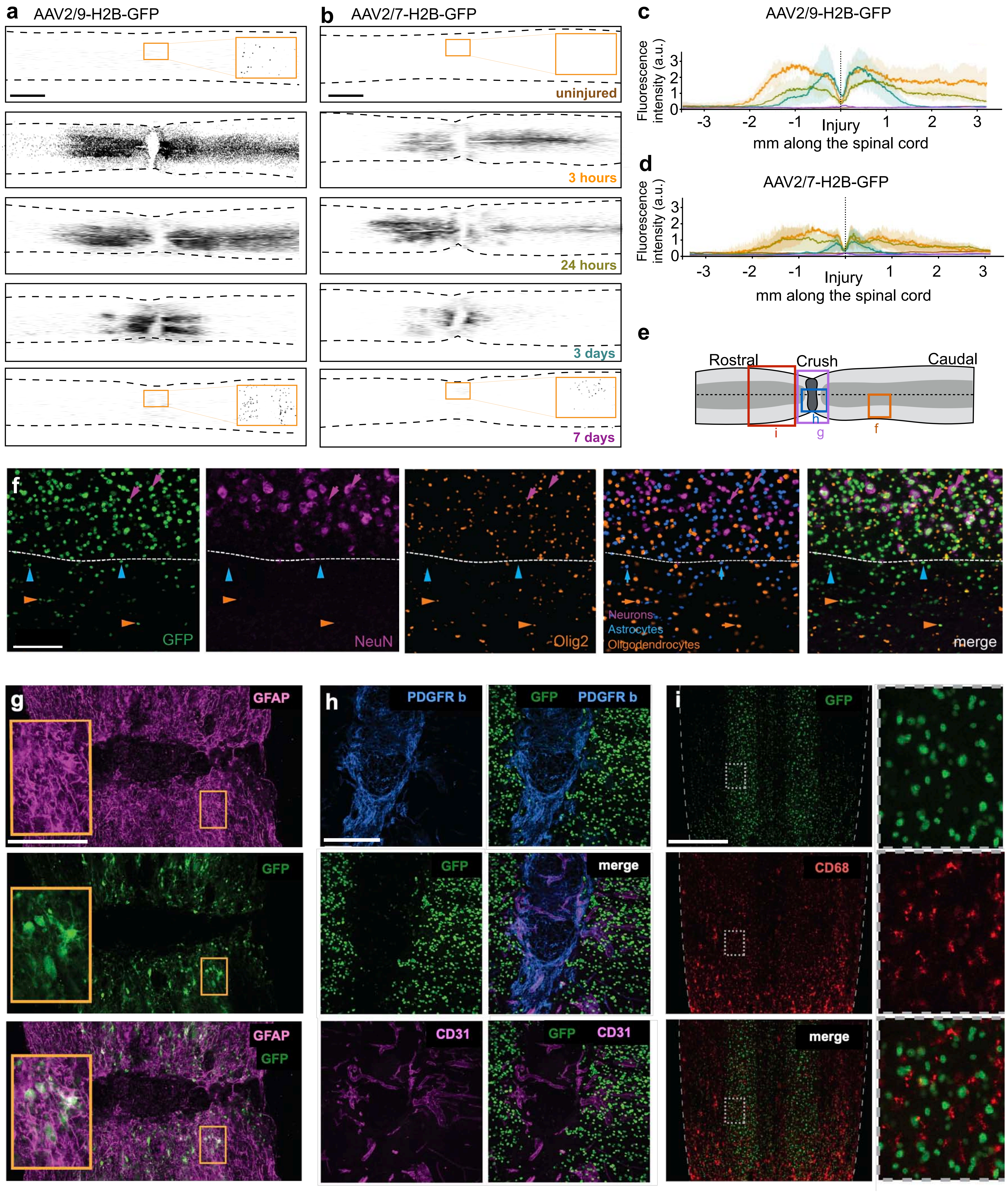

Supplementary Figure 1

Supplementary Fig. 1. Tropism of intravenously injected AAVs in the injured spinal cord acutely following T8 crush injury. (a, b) Representative images of longitudinal sections of the spinal cord infected with AAV2/9-CAG-H2B-GFP **(a)** or AAV2/7-CAG-H2B-GFP **(b)** through tail vein injection before, or 3 h, 24 h, 3 d, or 7 d after a T8 crush. Similar distributions were observed in all of 3 analysed mice for each time point. Scale bar: 1 mm. **(c, d)** Quantification of GFP expression in AAV2/9 and AAV2/7 groups. Mean ± SD is indicated by line and shadow with corresponding colour. a.u.: arbitrary unit. The fluorescence intensities were normalized to that in AAV2/9 injected at 3h after injury (n = 3 mice, 5 sections per mouse spanning the ventral to dorsal axis). **(e)** Schematic of image locations shown in **f-i**. **(f)** Colocalization of AAV2/9-expressed H2B-GFP (green) with markers for neurons (NeuN, magenta) and oligodendrocytes/OPCs (Olig2, orange). AAV2/9-H2B-GFP signal, pseudo-coloured to indicate cells co-labeled with NeuN (neurons, magenta), Olig2 (oligodendrocytes/OPCs, orange), and for cells absent of NeuN or Olig2 (presumptive astrocytes, blue). Scale bar: 100 µm. **(g)** Co-labeling of infected cells (cytosolic GFP, green) with an astrocyte marker (GFAP, magenta). Scale bar: 300 µm. **(h)** Co-staining of transduced cells (H2B-GFP, green) with markers for endothelial cells (CD31, magenta) and pericytes (PDGFRβ, blue). Scale bar: 300 µm. **(i)** Co-labeling of infected cells (H2B-GFP, green) with a macrophage/microglia marker (CD68, red). Scale bar: 300 µm. Similar distributions were observed in all of 3 analysed mice per serotype in f-i.

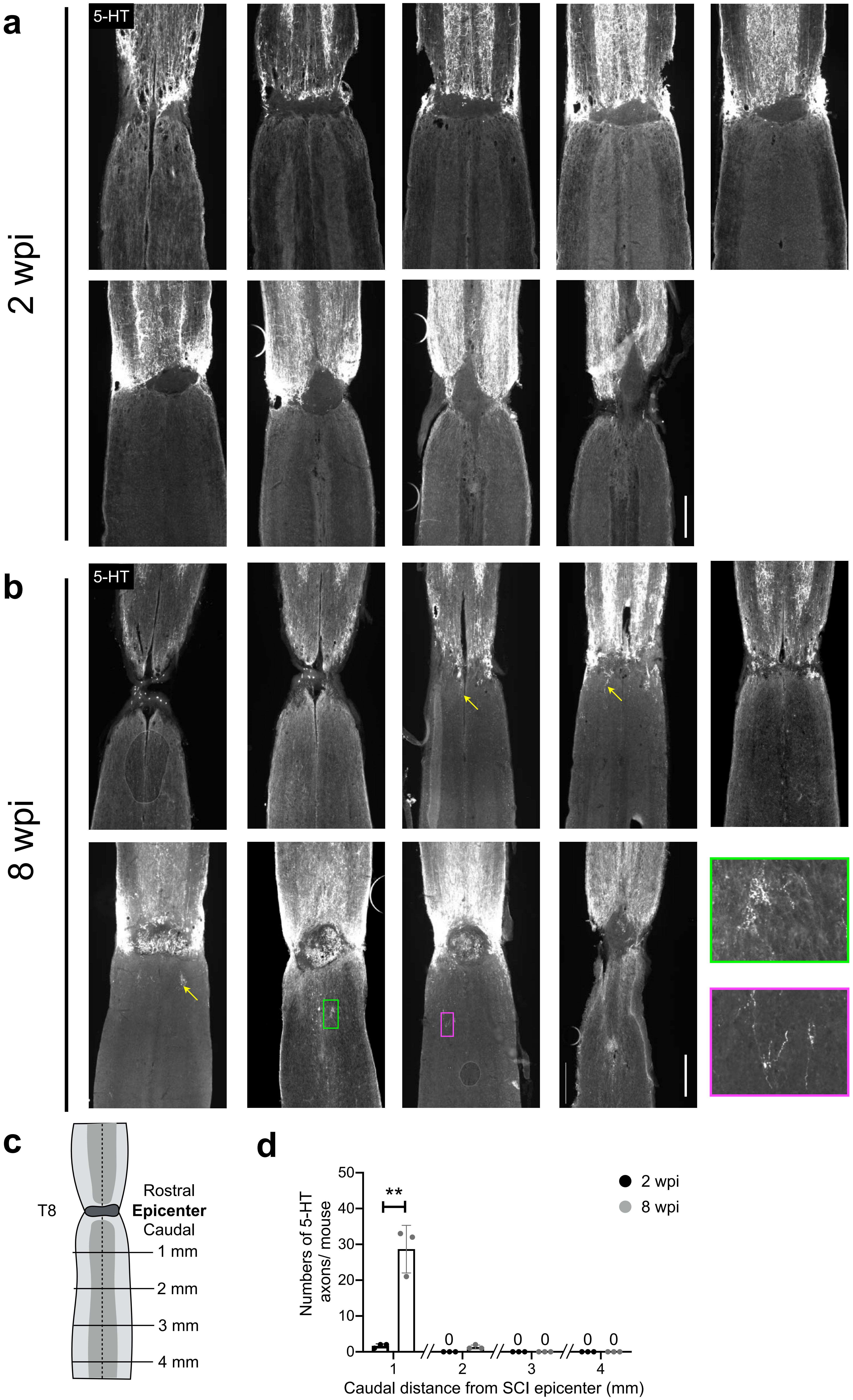

Supplementary Figure 2

Supplementary Fig. 2. **Characterization of T8 complete crush injury. (a, b)** Images of the serial sections (every four 40 µm sections) stained with anti-5-HT antibodies from mice at 2 weeks **(a)** or 8 weeks **(b)** after injury. Scale bar: 500 µm. Enlarged images of boxed area show the regrowth of 5-HT fibres. **(c, d)** Scheme **(c)** and result **(d)** of quantification of the number of 5-HT axons per mouse in the spinal cord distal to the lesion site from serial sections spanning the ventral to dorsal axis. Data shown as mean ± SD. 1mm: Two-tailed two sample t-test; 2mm: Wilcoxon rank sum test; $^{**}p = 0.0022$ ($n = 3$ mice, 9 sections per mouse).

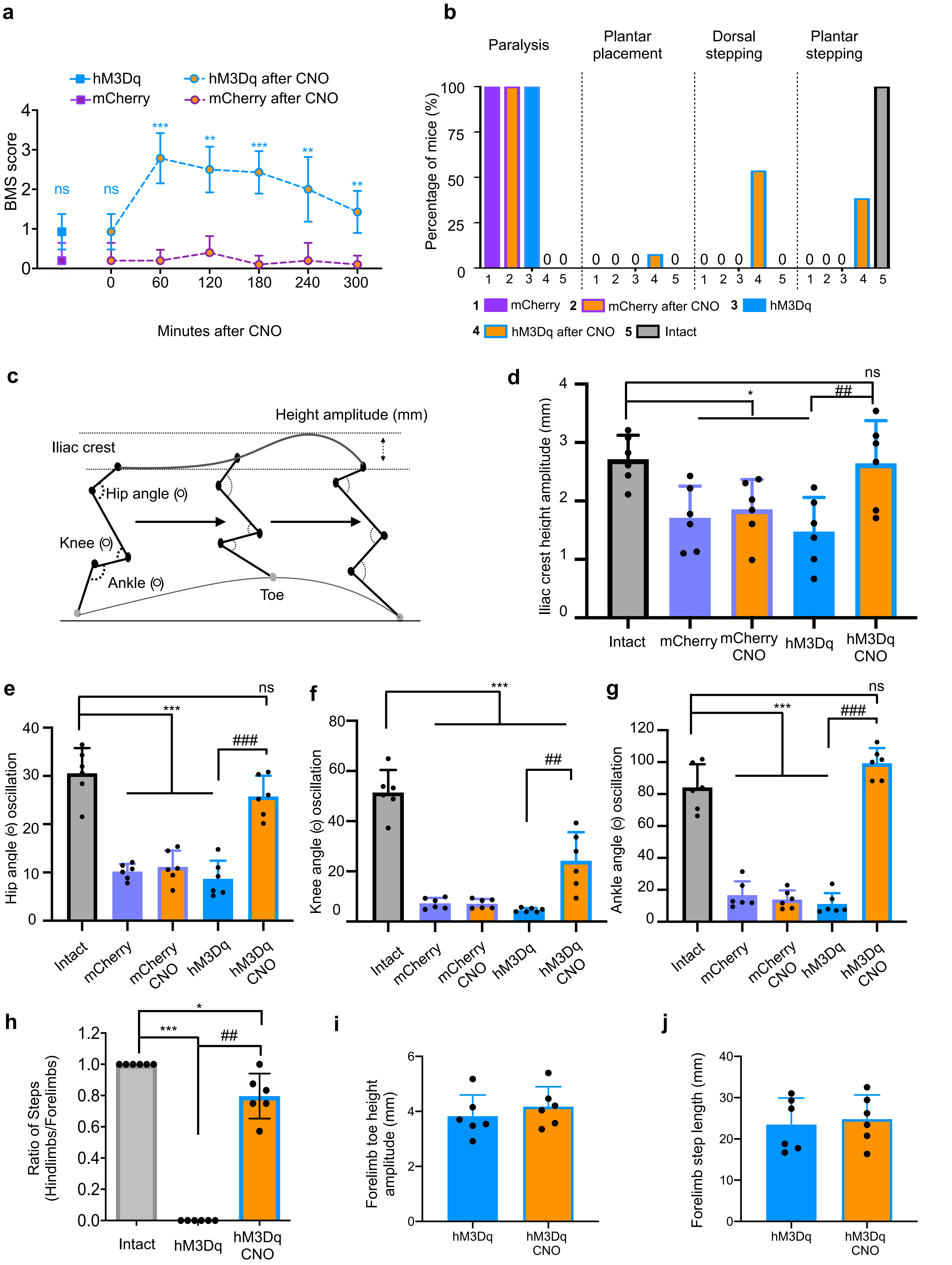

Supplementary Figure 3

Supplementary Fig. 3. Kinematic results of hindlimb locomotion in injured mice with hM3Dq-assisted activation of thoracic neurons. (a) BMS scores of mice (4 weeks after injury) with hM3Dq or control AAVs at indicated time points before and after CNO (1 mg/kg) injection. n = 7 (hM3Dq) and n = 5 (mCherry). Data shown as mean ± SD (also for the other panels of this figure). Repeated measures ANOVA followed by Bonferroni's post hoc. ns: no statistical difference, $p = 0.154$ (before CNO); $p = 0.154$ (0 min); ***$p < 0.0001$ (60 min, 180 min); **$p = 0.0002$ (120 min); 0.0050 (240 min) and 0.002 (300 min)**. (b)** Breakdown of behavioural performance categories. **(c)**, a simplified stickview to show one step cycle of one hindlimb at free walking. The arrow depicts the direction of the movement and the measurement of different ankle joints and iliac crest height amplitude are also shown. **(d-g)** Quantification of average iliac crest height amplitude (**d**) and average angle oscillations of hip (**e**), knee (**f**) and ankle (**g**) of mice expressing excitatory hM3Dq before and after CNO (1 mg/kg) in comparison to intact mice. n = 6 for each group. One-Way ANOVA with Tukey's post test for between-group (*) and two tailed paired t-tests for within-group (#) comparison. **(d)** *$p = 0.011$ (mCherry), 0.04 (mCherry CNO), 0.010 (hM3Dq), ##$p = 0.0088$, ns: no statistical difference, $p = 0.9746$; **(e)** ***/###$p < 0.001$, ns $p = 0.1717$; **(f)** ***$p < 0.001$, ##$p = 0.0079$; **(g)** ***/###$p < 0.001$,ns $p = 0.0622$. **(h)** Quantification of stepping frequencies of forelimbs and hindlimbs of WT mice with hm3Dq expression before and after CNO at 1 mg/kg in comparison with intact mice. Data shown as mean ± SD. Two tailed sign test was used for within-group comparison (#) and Wilcoxon rank sum were used for between-group comparison (*). ##$p = 0.0022$; ***$p < 0.001$; *$p = 0.0152$. n = 6 for each group. **(i, j)** Quantification of average forelimbs toe height amplitude **(i)** and forelimbs step length **(j)** in mice expressing excitatory hM3Dq before and after CNO in comparison to intact mice. Two tailed paired t-test. n= 6 for each group.

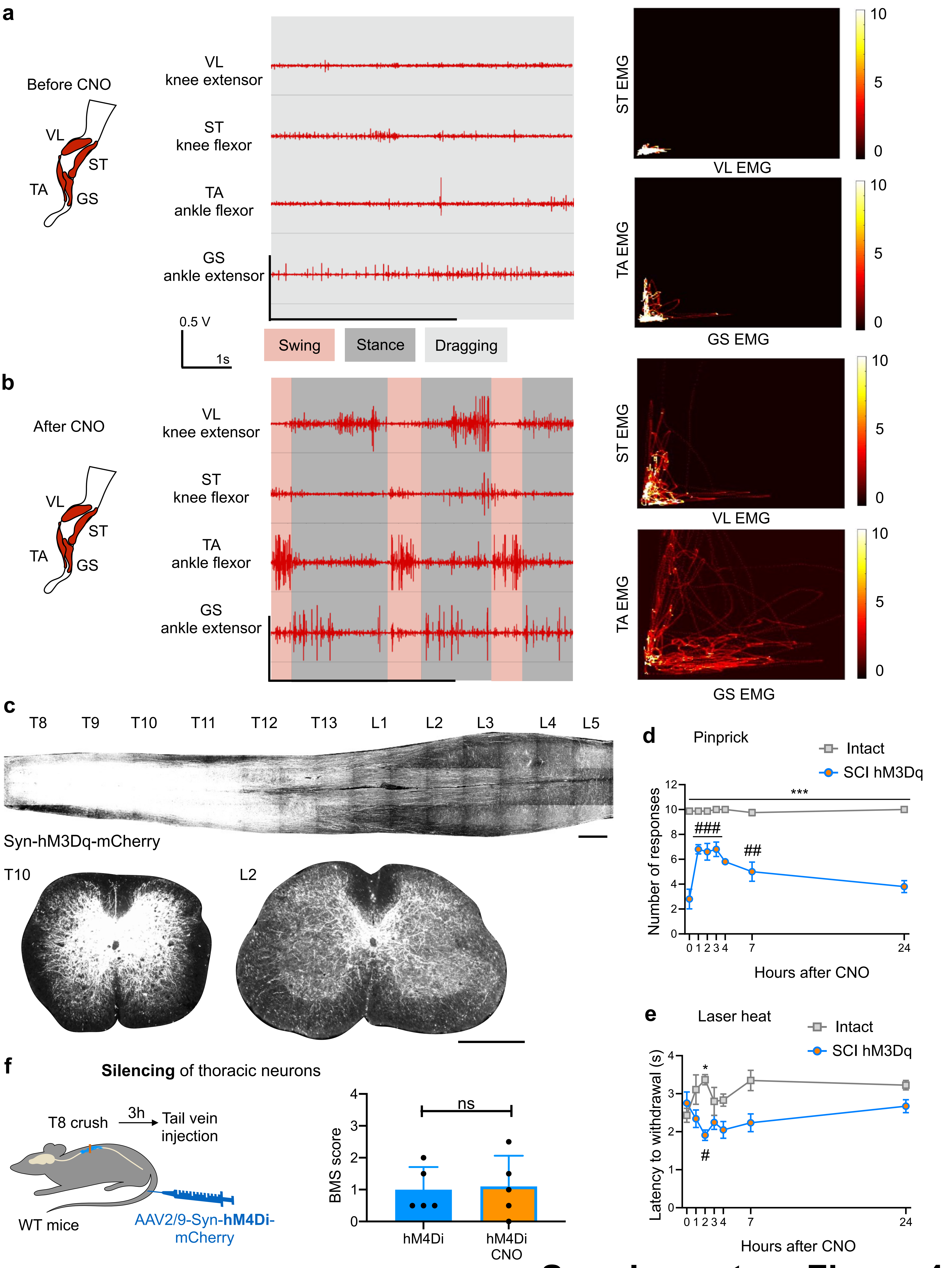

Supplementary Figure 4

Supplementary Fig. 4. hM3Dq-mediated activation of thoracic neurons elicits locomotion after T8 crush. (a, b) Left panels: EMG of selected muscles of the hind limbs in mice with hM3Dq expression before **(a)** and after **(b)** CNO (1mg/kg). Right panels: Probability density distributions of normalized EMG amplitudes in selected muscles during free walking. n=3 mice, 6-10 steps/mouse. **(c)** Representative images of in spinal cord sections from a wild type mouse with T8 crush and tail vein injection of AAV-Syn-hM3Dq-mCherry, n=8. Scale bars: 500 µm. **(d, e)** Quantification of number of responses after pinprick stimulation **(d)** or latency to withdraw after laser heat stimulation **(e)** Pinprick stimuli were applied 10 times per mouse. CNO administration concentration: 1mg/kg. Laser heat was applied to plantar surface of either hindlimb and the average time to withdrawal of 3 trials was measured. n = 5 (intact) and n = 8 (SCI). Anova with repeated measures followed by Bonferroni's post hoc for comparing intact vs SCI hMD3q (*) or different time points of SCI hMD3q (#). ##p = 0.0071; ***/###p < 0.001; #p = 0.0103. Data shown as mean ± SD**. (f)** Experimental outline and BMS scores of mice that received tail vein injection of inhibitory hM4Di at 3h after crush. Data shown are mean ± SD. Two-tailed paired t-test. ns: no statistical difference. n = 5.

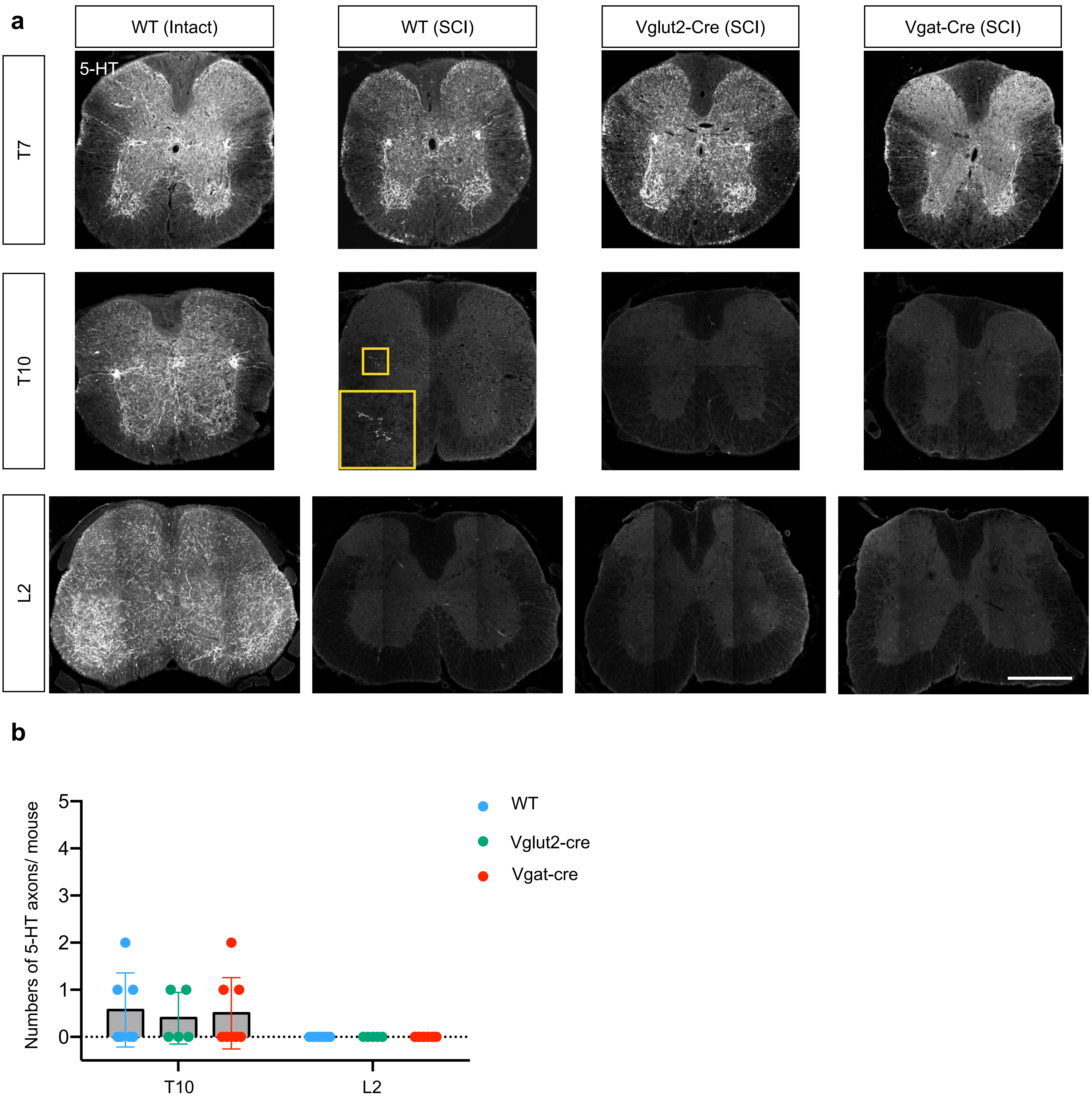

Supplementary Fig. 5. Verification of complete T8 crush injury in different groups. (a) Representative images showing serotonergic axons at T7, T10 or L2 of different groups of mice used in this study. Scale bars: 500 μm. **(b)** Quantification of 5-HT axon numbers from cross sections at T10 and L2 levels from WT, Vglut2-Cre, and Vgat-Cre mice at 8 weeks after injury. Data shown as mean ± SD. n = 7 (WT mice), n = 5 (vGlut2-Cre mice), n = 8 (Vgat-Cre mice). Less than 2 axons were seen at T10 and none was seen at L2 per mouse.

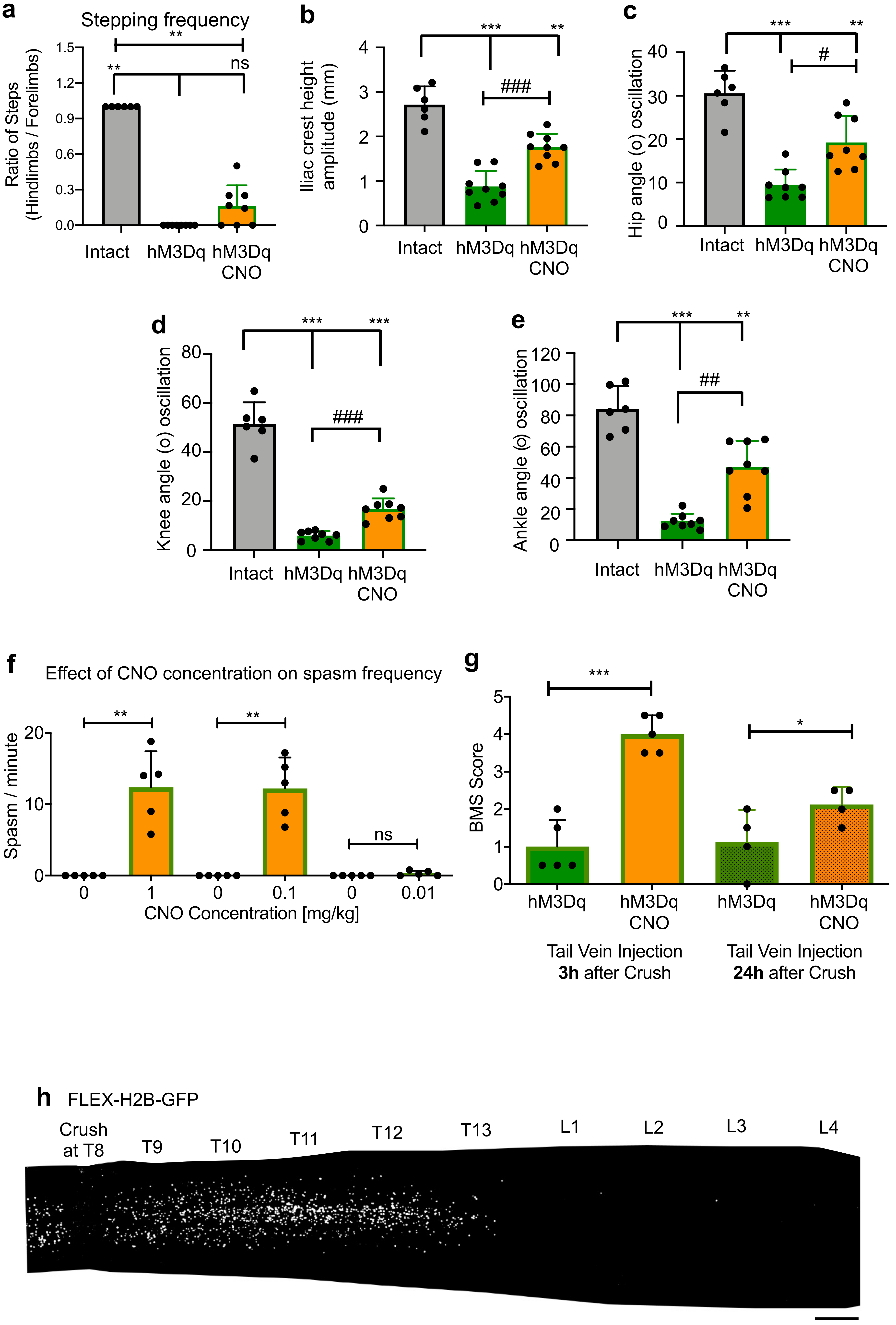

Supplementary Figure 6

Supplementary Fig. 6. Activation of thoracic excitatory neurons improves standing but only minimal stepping in mice with T8 crush injury. (a) Quantification of stepping frequencies of Vglut2-Cre mice with expression of hM3Dq in thoracic excitatory neurons after administration of CNO (0.01 mg/kg), in comparison with intact mice. Data shown as mean ± SD for all panels in this figure. Two-tailed paired t-test was used for within-group comparison and Wilcoxon rank sum was used for between-group comparison (*). **p = 0.0002 (hM3Dq), 0.0004 (hM3Dq CNO). n=6 (intact) and n = 8 (SCI). **(b-e)** Quantification of average amplitude of illiac crest heights **(b)** and average oscillation of hip **(c)** , knee (**d)** and ankle (**e**) joints**.** Two-tailed paired t-test was used for within-group (#) and 2 sample t-test was used for between group comparison (*). **(b)** ***/###p < 0.0001, **p = 0.0078; **(c)** ***p < 0.0001, **p = 0.004; #p = 0.028; **(d)** ***/###p < 0.0001, **(e)** ***p < 0.0001,**p = 0.006, ##p = 0.004. n = 6 (Intact) and n = 8 (hM3Dq). Data from intact mice are replotted from Figure S3c, d for comparison. **(f)** Quantification of spasms episodes in Vglut2-Cre mice with expression of excitatory hM3Dq in thoracic excitatory neurons after administration of indicated concentrations of CNO (n = 5). Two-tailed paired t-test; **p = 0.0054 (1 mg/kg); 0.0033 (0.1 mg/kg); no statistical difference (ns) p = 0.1202. **(g)** BMS scores (before and after CNO at 0.01mg/kg) of Vglut2-Cre mice with tail vein injection of AAV2/9-Syn-FLEX-hM3Dq at 3h or 24h after crush (3h data are replotted from Figure 3 for comparison). Two tailed paired t-test; ***p < 0.0001; *p = 0.0388. n= 5 (3h) and n = 4 (24h). **(h)** A representative image showing the distribution of transduced neurons in different spinal levels from Vglut2-Cre mice receiving tail vein injection of AAV2/9-CAG-FLEX-H2B-GFP at 3 hours after crush. Similar distributions were observed in all of 3 analysed mice. Scale bar: 1mm.

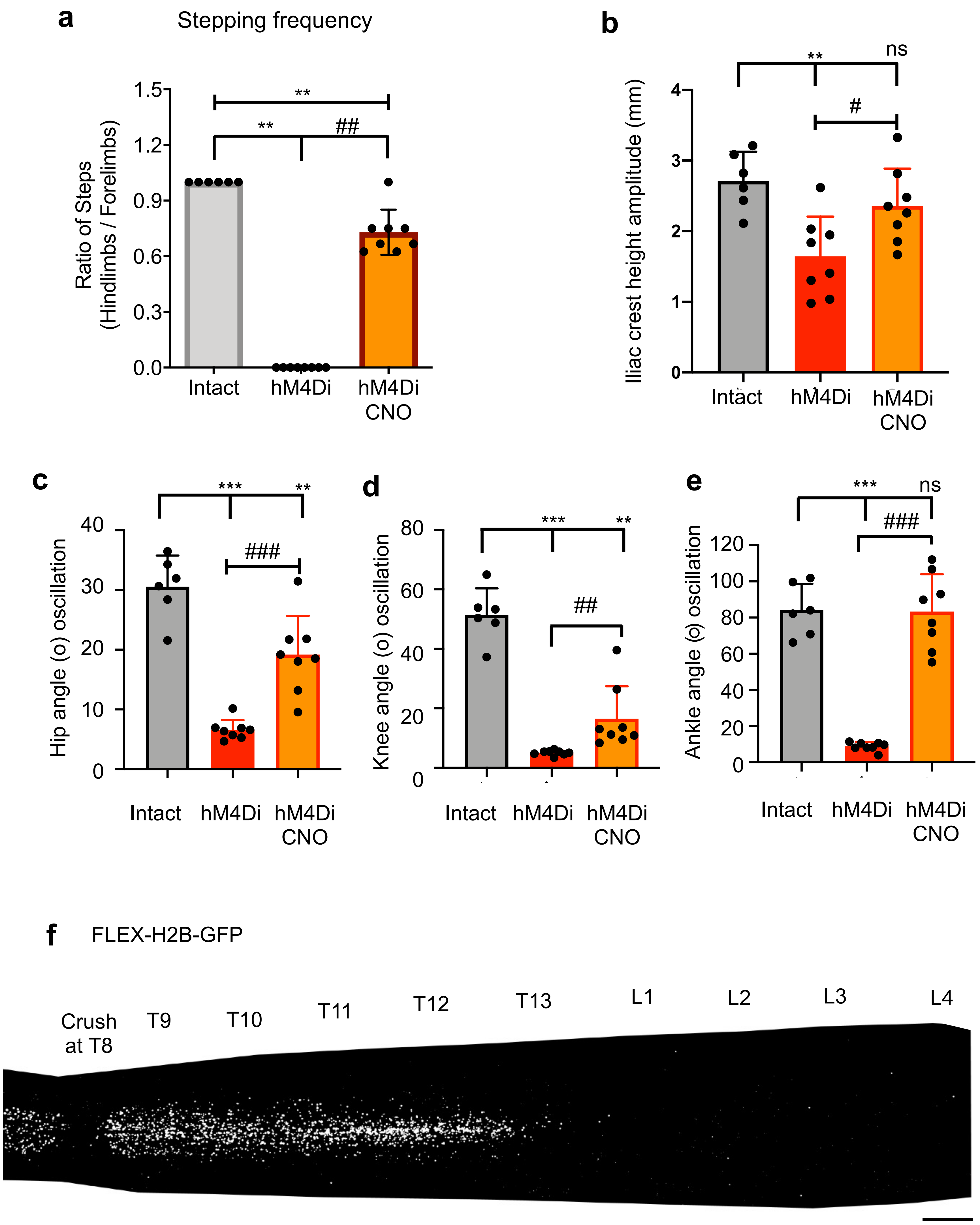

Supplementary Figure 7

Supplementary Fig. 7. Inhibition of thoracic inhibitory neurons preferentially improves stepping ability in mice after T8 crush injury. (a) Quantification of stepping frequencies of forelimbs and hindlimbs of Vgat-Cre mice with hM4Di expression before and after CNO at 5 mg/kg in comparison with intact mice. Data from intact mice are replotted from Supplementary Fig. S6a for comparison. (n=6 and 8 for intact and hM4Di respectively). Data shown as mean ± SD (also for other panels of this figure). Two-tailed sign test was used for within-group comparison (#) and Wilcoxon rank sum were used for between-group comparison (*); **p = 0.0033; ***p < 0.001; ##p = 0.0078. **(b-e)** Quantification of average amplitude of illiac crest heights **(b)** and average oscillation of hip **(c)** knee **(d)** and ankle **(e)** joints. Two-tailed paired t-test was used for within-group comparison (#) and 2 sample ttest was used for between-group comparison (*). **(b)** **p = 0.004, #p = 0.0106, ns p = 0.5913; **(c)** ***/###p < 0.0001, **p = 0.0043; **(d)** ***p < 0.001, **p = 0.0013, ##p = 0.0078; **(e)** ***/###p < 0.001, n.s. p = 0.9352. n = 6 (Intact), n = 8 (hM4Di). Data from intact mice from Figure S3c, d are replotted here for comparison. **(f)** A representative image showing distribution of GFP+ cells in different spinal levels from Vgat-Cre mice receiving tail vein injection of AAV2/9-CAG-FLEX-H2B-GFP after crush. Similar distributions were observed in all of 3 analysed mice. Scale bar: 1mm.

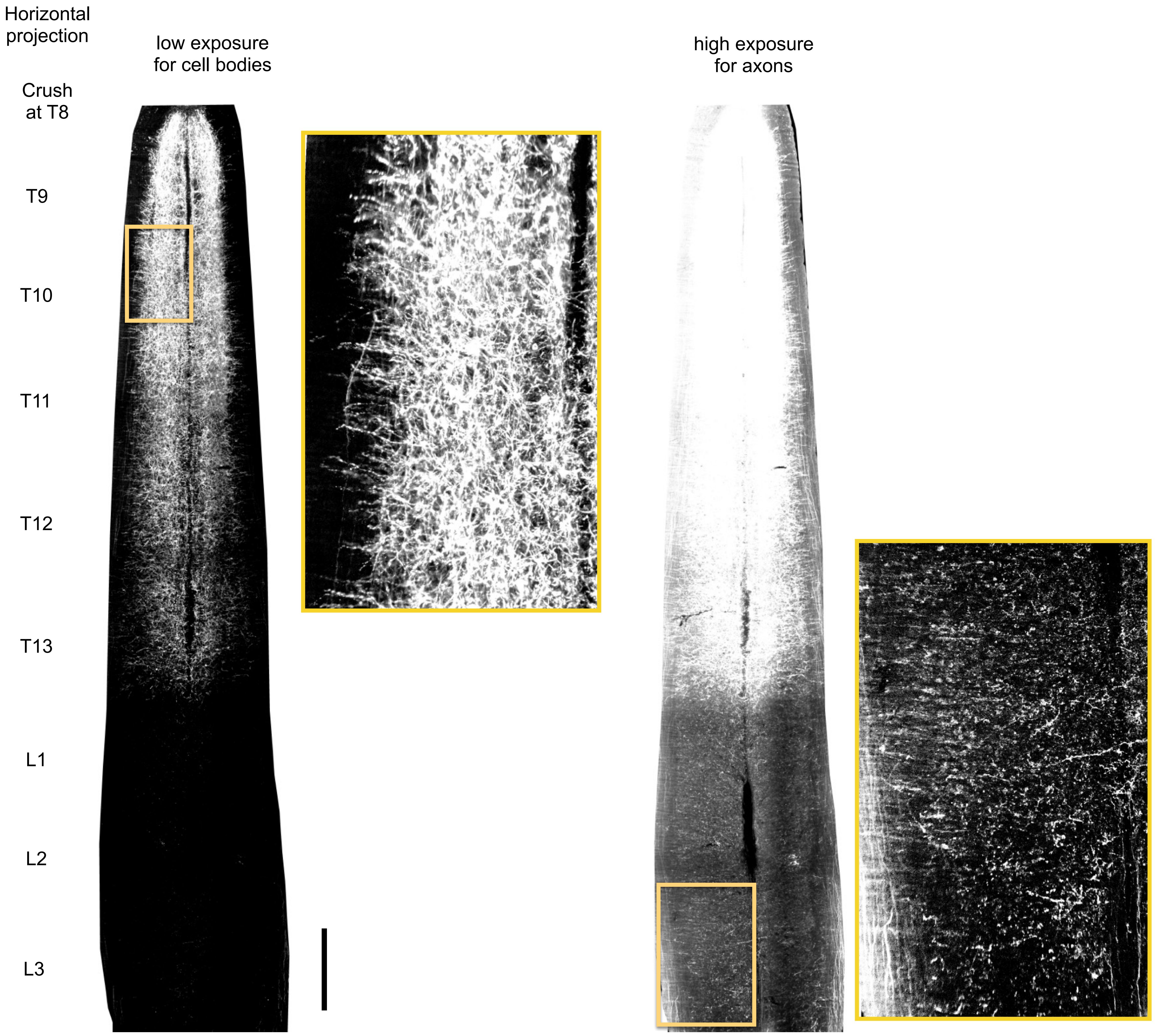

Supplementary Fig. 8. Histological data showing labelled neurons and axons by intersectional targeting of hM3Dq-mCherry in wild type mice. Representative images of longitudinal spinal sections showing the distribution of mCherry+ neurons and axons at low (left) and high exposure, in a wild-type mouse with intersectional targeted expression of hM3Dq-mCherry. Enlarged images of boxed area show the soma (low exposure) and axon projections (high exposure). Scale bars: 1mm, n=3.

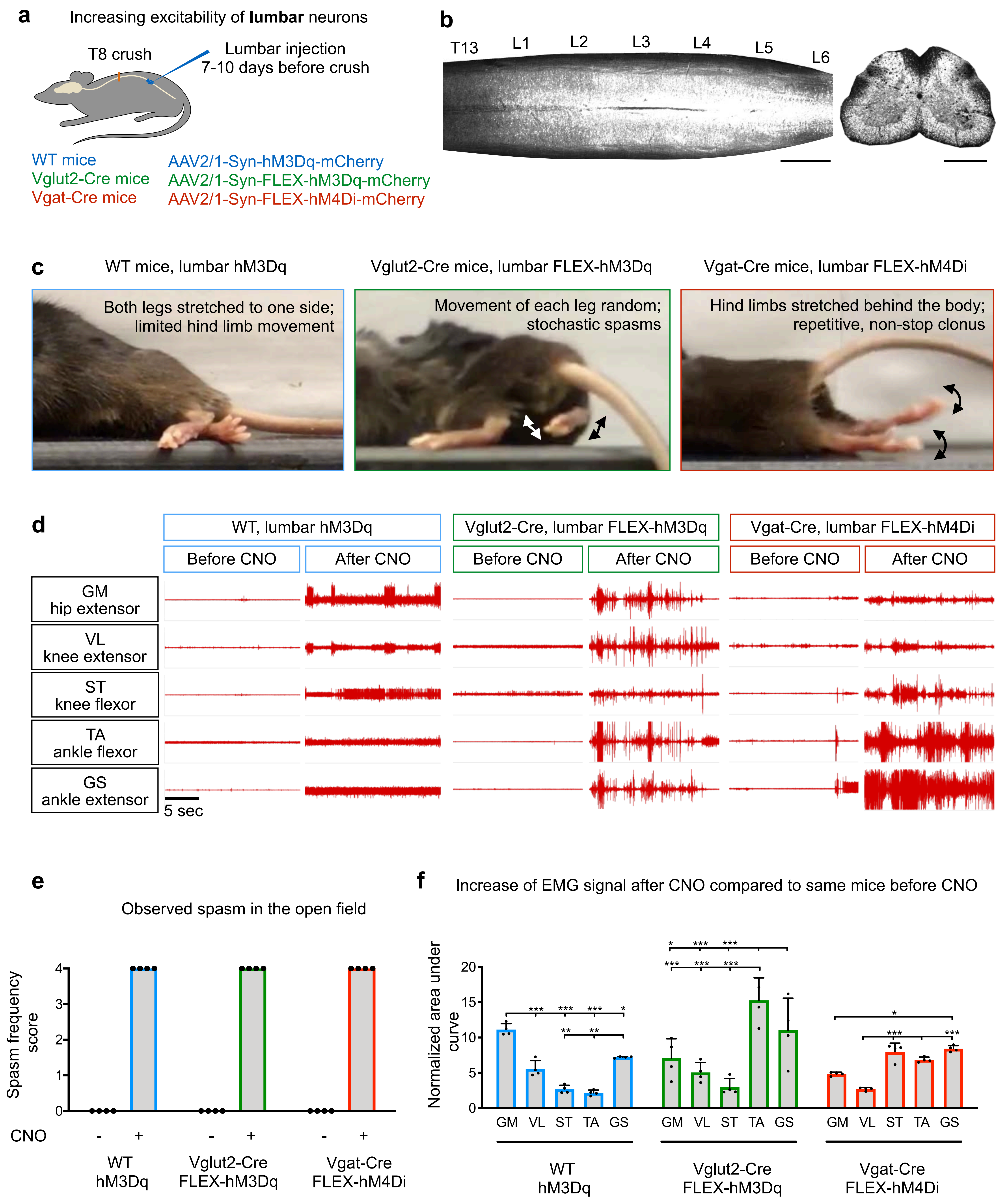

Supplementary Figure 9

Supplementary Fig. 9. Manipulations of excitatory and/or inhibitory neurons in the lumbar spinal cord leads to different forms of spasms. (a) Experimental Schematic. WT, Vglut2-Cre and Vgat-Cre mice (n = 4 per group) received a lumbar spinal cord injection of AAV2/1 versions of corresponding DREADDs 7-10 d before a T8 crush. **(b)** Representative images of longitudinal and cross spinal sections showing the distribution of mCherry+ neurons and axons. Similar patterns were observed in 3 different mice per group. Scale bars: 1mm (longitudinal section) and 500 μm (cross section). **(c)** Images showing representative depictions of muscle spasms observed in each group. **(d)** Representative EMG recordings of hip extensor, knee flexor and extensor, and ankle flexor and extensor before and after CNO in each group (WT mice: 1 mg/kg, Vglut2-Cre: 0.01 mg/kg, Vgat-Cre: 5 mg/kg). **(e)** Quantification of spasm frequencies in indicated groups of mice with CNO treatment. n = 4 for each group. **(f)** Quantification of EMG signals showing area under curve for indicated groups of mice after CNO, normalized with same mice before CNO application. Shown are means ± SD. One-Way ANOVA with Tukey's post test; ns: no statistical difference, WT hM3Dq: *: p= 0.0229, **: p= 0.0058 (ST), 0.0018(TA), ***: p < 0.0001, vGlut2-cre FLEX-hM3Dq: *: p=0.0439, ***: p < 0.0001; Vgat-Cre FLEX-hM4Di: *:p= 0.0439, ***: p < 0.000. n = 4 for each group.

Supplementary table

Primer name	Primer sequence
WPRE-forward	CACTGACAATTCCGTGGTGT
WPRE-reverse	GAGATCCGACTCGTCTGAGG
ITR-forward	GACCTTTGGTCGCCCGGCCT
ITR-reverse	GAGTTGGCCACTCCCTCTCTGC

Supplementary table. Primers used for quantification of viral titres.
