# Supplementary material for: Improving hindlimb locomotor function by Non-invasive AAV-mediated manipulations of propriospinal neurons in mice with complete spinal cord injury
Source: Nat Commun. 2021 Feb 3;12:781. doi: 10.1038/s41467-021-20980-4 (PMC7859413; doi:10.1038/s41467-021-20980-4)
Supplement: Supplementary file 6 — Description of additional supplementary files [file 41467_2021_20980_MOESM6_ESM.docx]

Description of additional supplementary information files

Title: Supplementary Movie 1.

Description: Wild type mice expressing excitatory DREADD in the thoracic cord around the lesion site.

Title: Supplementary Movie 2.

Description: vGlut2-Cre mice expressing excitatory DREADD in excitatory neurons in the thoracic cord around the lesion site.

Title: Supplementary Movie 3.

Description: Vgat-cre mice expressing inhibitory DREADD in the in inhibitory neurons in the thoracic cord around the lesion site.

Title: Supplementary Movie 4.

Description: Mice expressing DREADD in the lumbar spinal cord
