# Supplementary material for: Improving hindlimb locomotor function by Non-invasive AAV-mediated manipulations of propriospinal neurons in mice with complete spinal cord injury
Source: Nat Commun. 2021 Feb 3;12:781. doi: 10.1038/s41467-021-20980-4 (PMC7859413; doi:10.1038/s41467-021-20980-4)
Supplement: Supplementary file 7 — Reporting Summary [file 41467_2021_20980_MOESM7_ESM.pdf]

nature research

Corresponding author(s): Bo Chen, Zhigang He
Last updated by author(s): Dec 1, 2020

Reporting Summary

Nature Research wishes to improve the reproducibility of the work that we publish. This form provides structure for consistency and transparency in reporting. For further information on Nature Research policies, see our Editorial Policies and the Editorial Policy Checklist.

Statistics

For all statistical analyses, confirm that the following items are present in the figure legend, table legend, main text, or Methods section.

n/a	Confirmed	
☐	☒	The exact sample size (n) for each experimental group/condition, given as a discrete number and unit of measurement
☐	☒	A statement on whether measurements were taken from distinct samples or whether the same sample was measured repeatedly
☐	☒	The statistical test(s) used AND whether they are one- or two-sided<br>*Only common tests should be described solely by name; describe more complex techniques in the Methods section.*
☐	☒	A description of all covariates tested
☐	☒	A description of any assumptions or corrections, such as tests of normality and adjustment for multiple comparisons
☐	☒	A full description of the statistical parameters including central tendency (e.g. means) or other basic estimates (e.g. regression coefficient) AND variation (e.g. standard deviation) or associated estimates of uncertainty (e.g. confidence intervals)
☐	☒	For null hypothesis testing, the test statistic (e.g. F, t, r) with confidence intervals, effect sizes, degrees of freedom and P value noted<br>*Give P values as exact values whenever suitable.*
☒	☐	For Bayesian analysis, information on the choice of priors and Markov chain Monte Carlo settings
☒	☐	For hierarchical and complex designs, identification of the appropriate level for tests and full reporting of outcomes
☒	☐	Estimates of effect sizes (e.g. Cohen's d, Pearson's r), indicating how they were calculated

Our web collection on statistics for biologists contains articles on many of the points above.

Software and code

Policy information about availability of computer code

Data collection	Imaging data was collected using Zeiss 700 or Zeiss 710, and Zen Blue for controlling the microscope Zeiss LSM710<br>Behavior data was collected using iphone6.<br>Eletrophysiology data was collected using AC amplifier (1700, A-M Systems), a digitizer (PowerLab 16/35, ADInstruments)
Data analysis	Immunostaining for cell number counts was measured using ImageJ (v.2.0.0) software.<br>Statistic analysis was performed by GraphPad Prism (9), Microsoft Excel(2019), SAS (v9.4),<br>Figures were generated by Prism (9).<br>EMG data was analyzed by LabChart 8, and MATLAB (R2018).<br>Behavior data was analyzed by Deeplabcut and Custom-written codes for MATLAB (R2018).

For manuscripts utilizing custom algorithms or software that are central to the research but not yet described in published literature, software must be made available to editors and reviewers. We strongly encourage code deposition in a community repository (e.g. GitHub). See the Nature Research guidelines for submitting code & software for further information.

Data

Policy information about availability of data

All manuscripts must include a data availability statement. This statement should provide the following information, where applicable:

- Accession codes, unique identifiers, or web links for publicly available datasets
- A list of figures that have associated raw data
- A description of any restrictions on data availability

The data supporting the findings of this study are available within the paper and its supplementary information files. Datasets generated and/or analyzed in the current study are provided as separate Source Data for Fig.1b,Fig.2b,2d,2e, 2f, 2g, Fig.3b, 3d,3e,3f,Fig.4b, 4d, 4e, 4f, Fig.5c,5d,5e, Supplementary Fig.1c, 1d,

Supplementary Fig.2d, Supplementary Fig. 3a,3b,3d,3e,3f, 3g, 3h,3i,3j, Supplementary Fig.4d,4e,4f, Supplementary Fig.5b, Supplementary Fig.6b,6c, 6d,6e,6f,6g, Supplementary Fig.7a,7b,7c,7d,7e,Supplementary Fig.9e and 9f. Other data generated during and/or analyzed during the current study are available from the corresponding author on reasonable request.

Field-specific reporting

Please select the one below that is the best fit for your research. If you are not sure, read the appropriate sections before making your selection.

☒ Life sciences ☐ Behavioural & social sciences ☐ Ecological, evolutionary & environmental sciences

For a reference copy of the document with all sections, see nature.com/documents/nr-reporting-summary-flat.pdf

Life sciences study design

All studies must disclose on these points even when the disclosure is negative.

Sample size	Sample sizes were indicated in the legend of each Figure and Supplementary Figure. No statistical methods were used to predetermine sample size. Estimates were made based on our previous experience, experimental approach, availability and feasibility required to obtain statistically significant results.
Data exclusions	In most of experiments, we included all data. For mice with injured hindlimbs (loss of toes or swollen paw) after SCI were excluded from behavior tests.
Replication	The experimental findings were reliably reproduced, for representative data used for statistical analysis, the number of animals or experiments is described in corresponding figure legends.
Randomization	Different groups are mainly based on different genotypes of mice, which excludes the possibility / necessity of randomization.<br>For WT mice, mice were randomly selected to receive DREADD or control virus.
Blinding	All surgeries were carried out blinded to the genotype/treatment of the mice during the experiments. For EMG recoding, behavior and image analysis were quantified in a blinded manner.

Reporting for specific materials, systems and methods

We require information from authors about some types of materials, experimental systems and methods used in many studies. Here, indicate whether each material, system or method listed is relevant to your study. If you are not sure if a list item applies to your research, read the appropriate section before selecting a response.

Materials & experimental systems

n/a	Involved in the study	
☐	☒	Antibodies
☒	☐	Eukaryotic cell lines
☒	☐	Palaeontology and archaeology
☐	☒	Animals and other organisms
☒	☐	Human research participants
☒	☐	Clinical data
☒	☐	Dual use research of concern

Methods

n/a	Involved in the study	
☒	☐	ChIP-seq
☒	☐	Flow cytometry
☒	☐	MRI-based neuroimaging

Antibodies

Antibodies used	Primary antibodies used were: chicken anti-GFP (1:1000, Abcam ab13970); rabbit anti-RFP (1:500, Abcam ab34771); mouse anti-NeuN (1:500, Millipore MAB377); rabbit anti-GFAP (1:500, Dako Z0334); rabbit anti-Olig2 (1:300, Millipore AB9610); rabbit anti-PDGFRβ (1:500, Thermo Fisher Scientific MA5-15143); rat anti-CD68 (1:500, BioRad MCA1957); rat anti-CD31 (1:1:500, BD Biosciences 550274); and goat anti-5-HT (1:200, Immunostar 20079).<br>The following fluorescent secondary antibodies were used at a dilution of 1:400: 488 Donkey anti-Chicken (703-545-155), 488 Donkey anti-Rabbit (711-545-152), Biotin Donkey anti-Rabbit (711-066-152), HRP Donkey anti-Rabbit (715-035-151), Biotin Donkey anti-Rat (712-065-153), HRP Donkey anti-Mouse (712-035-153), Biotin Donkey anti-Mouse (715-065-151), HRP Donkey anti-Mouse (715-035-150) from Jackson ImmunoResearch, 568 Donkey anti-Rabbit (A10042) and 647 Donkey anti-Goat (A-21447) from Thermo Fisher Scientific (Invitrogen) and 647 Streptavidin (S-21374) and 568 Streptavidin (S-11226) from Life Technologies.
Validation	For anti-5-HT, anti-GFAP, anti-GFP, anti-RFP and secondary antibodies see Ref. 22 in this paper; For anti-CD68 and anti-CD31, see Dias et al., Cell. 2018 173 1-13.

Animals and other organisms

Policy information about studies involving animals; ARRIVE guidelines recommended for reporting animal research

Laboratory animals	Wild-type C57BL/6 mice were purchased from Charles River Laboratories. Vglut2-Cre and Vgat-Cre mice were obtained from Jackson Laboratory (Jax stock # 016963 and 028862, respectively). Surgeries were conducted on adult female mice (18-21 g) at the age of 8-10 weeks. Mice were given ad libitum access to food and water, and housed in cages under 12h day/night cycles with bedding changed frequently. Mice were not permitted to breed before or during their inclusion in in vivo experiments.
Wild animals	The study did not involve wild animals.
Field-collected samples	The study did not involve samples collected from the field.
Ethics oversight	All animal experiments were performed in compliance with protocols approved by the IACUC at Boston Children's Hospital.

Note that full information on the approval of the study protocol must also be provided in the manuscript.
